# Effects of gene mutation and disease progression on representative neural circuits in familial Alzheimer’s disease

**DOI:** 10.1186/s13195-019-0572-2

**Published:** 2020-01-14

**Authors:** Meina Quan, Tan Zhao, Yi Tang, Ping Luo, Wei Wang, Qi Qin, Tingting Li, Qigeng Wang, Jiliang Fang, Jianping Jia

**Affiliations:** 1 Innovation Center for Neurological Disorders and Department of Neurology, Xuanwu Hospital, Capital Medical University, National Clinical Research Center for Geriatric Diseases, Beijing, People’s Republic of China; 2 Beijing Key Laboratory of Geriatric Cognitive Disorders, Beijing, People’s Republic of China; 3 Clinical Center for Neurodegenerative Disease and Memory Impairment, Capital Medical University, Beijing, People’s Republic of China; 4 Center of Alzheimer’s Disease, Beijing Institute for Brain Disorders, Beijing, People’s Republic of China; 5 Guang’anmen Hospital, China Academy of Chinese Medical Sciences, Beijing, China

**Keywords:** Familial Alzheimer’s disease, Neural circuits, Gene mutation, Diffusion tensor imaging, Resting-state functional MRI

## Abstract

**Background:**

Although structural and functional changes of the striatum and hippocampus are present in familial Alzheimer’s disease, little is known about the effects of specific gene mutation or disease progression on their related neural circuits. This study was to evaluate the effects of known pathogenic gene mutation and disease progression on the striatum- and hippocampus-related neural circuits, including frontostriatal and hippocampus-posterior cingulate cortex (PCC) pathways.

**Methods:**

A total of 102 healthy mutation non-carriers, 40 presymptomatic mutation carriers (PMC), and 30 symptomatic mutation carriers (SMC) of amyloid precursor protein (*APP*), presenilin 1 (*PS1*), or presenilin 2 gene, with T1 structural MRI, diffusion tensor imaging, and resting-state functional MRI were included. Representative neural circuits and their key nodes were obtained, including bilateral caudate-rostral middle frontal gyrus (rMFG), putamen-rMFG, and hippocampus-PCC. Volumes, diffusion indices, and functional connectivity of circuits were compared between groups and correlated with neuropsychological and clinical measures.

**Results:**

In PMC, *APP* gene mutation carriers showed impaired diffusion indices of caudate-rMFG and putamen-rMFG circuits; *PS1* gene mutation carriers showed increased fiber numbers of putamen-rMFG circuit. SMC showed increased diffusivity of the left hippocampus-PCC circuit and volume reduction of all regions as compared with PMC. Imaging measures especially axial diffusivity of the representative circuits were correlated with neuropsychological measures.

**Conclusions:**

*APP* and *PS1* gene mutations affect frontostriatal circuits in a different manner in familial Alzheimer’s disease; disease progression primarily affects the structure of hippocampus-PCC circuit. The structural connectivity of both frontostriatal and hippocampus-PCC circuits is associated with general cognitive function. Such findings may provide further information about the imaging biomarkers for early identification and prognosis of familial Alzheimer’s disease, and pave the way for early diagnosis, gene- or circuit-targeted treatment, and even prevention.

**Electronic supplementary material:**

The online version of this article (10.1186/s13195-019-0572-2) contains supplementary material, which is available to authorized users.

## Background

Alzheimer’s disease is the cause of 60–70% of cases of dementia, the population of which is estimated to be 40–50 million worldwide, more than doubled from 1990 to 2016 [1]. Without disease-modifying treatment, Alzheimer’s disease has become an increasing challenge to healthcare systems and economy in China [2] and internationally. Several recent clinical trials of Alzheimer’s disease targeting the amyloid hypothesis especially by lowering amyloid-beta (Aβ) have failed, which led us to think about other therapeutic targets for Alzheimer’s disease [3]. In recent years, circuit or network level treatments for AD have shown some promising results [4], such as deep-brain stimulation [5–7]. Following this trend, it is critical to look for circuit level imaging biomarkers that can help with early diagnosis and treatment.

Familial Alzheimer’s disease (FAD) accounts for 15–25% of total Alzheimer’s disease [8], and it has presented a useful model in studying pathogenesis and trajectory of the disorder. FAD includes autosomal dominant Alzheimer’s disease (ADAD), carrying known causative gene mutation including the amyloid precursor protein (*APP*), presenilin 1 (*PS1*), or presenilin 2 (*PS2*) that are nearly 100% certain to show sequential pre-dementia and dementia stage clinical features and thus can be diagnosed before symptoms onset. FAD also includes those carrying unknown variants in *PS1/PS2/APP* gene or other gene mutations. Our team has recently reported *PS1/PS2/APP* mutations in 404 Chinese pedigrees with FAD [9]. Treatment could also be initiated at a presymptomatic stage in FAD, which promotes the development of intervention and prevention trials [10, 11].

Imaging studies in FAD suggest that striatum and hippocampus are possibly the earliest involved brain regions. Amyloid positron emission tomography (PET) imaging studies in ADAD mutation carriers have shown striatal amyloid deposition before symptoms appeared [12–17], which were often greater than deposits observed in the signature regions such as medial temporal lobe and medial parietal and frontal cortices. A longitudinal imaging study of ADAD found elevated PET tracer uptake in all subcortical regions including the striatum, before the involvement of the hippocampus [18]. MRI studies confirmed the early volumetric changes of the striatum [15, 19, 20] and hippocampus [16, 21, 22] in FAD before symptom onset. In addition to volumetric changes, diffusion tensor imaging (DTI) confirmed finer structural changes of the striatum and hippocampus in preclinical stage of FAD relative to controls [20, 23]. There are also a few functional MRI studies showing early functional changes of the striatum or hippocampus in preclinical FAD [24–26]. These numerous imaging findings guided us to look for striatum- and hippocampus-related neural circuits.

The striatum receives cortical input projection fibers that are part of the glutamatergic neural circuits [27]. Subregions of the striatum, especially the superior and lateral part of caudate and putamen, receive dense projection from the dorsolateral prefrontal cortex (DLPFC), which plays a critical role in cognitive function such as executive function and working memory [28–33]. On the other hand, imaging studies found a significant relationship between hippocampus activation and posterior cingulate cortex (PCC) during episodic memory encoding and recognition task in mild cognitive impairment (MCI) patients [34] and association between hypometabolism in hippocampus-PCC subnetwork and episodic memory deficits in pre-dementia stage [35]. Notably, the above cognitive function domains on which the frontostriatal and hippocampus-PCC circuits play important roles are impaired even in the preclinical stage of Alzheimer’s disease [36, 37].

Up to now, little is known about the structural and functional features of frontostriatal and hippocampus-PCC neural circuits during the whole disease spectrum of FAD. Furthermore, most of the above imaging findings in FAD have involved mutation carriers of various genes, while not looked at the effect of specific gene. Imaging studies have shown gene- or mutation-specific effect on brain structure [38, 39] and function [40] in Alzheimer’s disease, but the gene-specific effect on the frontostriatal and hippocampus-PCC neural circuits remains uncertain.

This study was aimed at exploring the effects of specific gene mutations (primarily *PS1* and *APP*) and disease progression on the structure and function of frontostriatal and hippocampus-PCC circuits in FAD. We hypothesize that the structure and function of these representative circuits would be impaired in the early stages of FAD and would be more damaged with disease progression. Moreover, they would be affected differently by *PS1* and *APP* mutations.

## Methods

### Participants

All the participants were recruited from August 2014 to February 2018, as part of the ongoing Chinese Familial Alzheimer’s Disease Network study (CFAN, Study ID Number: SYXWJ002; ClinicalTrials.gov Identifier: NCT03657732) at Xuanwu Hospital, which receives research referrals from across China. Participants enrolled in CFAN study were examined on the basis of neuropsychological testing, neuroimaging, genetic testing, and fluid biomarker measurements. FAD in CFAN study was defined as at least one first-degree relative in addition to the patient himself/herself within the family who had an objective cognitive decline suggestive of AD [8]. We included 70 subjects carrying known gene mutations for this study. Forty of these subjects were presymptomatic mutation carriers (PMC), and the rest 30 subjects were symptomatic mutation carriers (SMC). The mutations include *PS1* (H163R, L282V, L392V, M270L, L271V, M139V, M139L, I213T, A285V, F105I, I100F, K311R, P433S, G111V, L173F, G206S), *PS2* (F181I, V214L, M298T, G34S), and *APP* (I716T, V717I, V715M). A group of 102 healthy mutation non-carriers (control) within the FAD pedigrees who do not carry the three known gene mutations were also included. All subjects underwent neuropsychological assessments including the Mini-Mental State Examination (MMSE) and the Montreal Cognitive Assessment (MoCA) for cognitive functions, and Clinical Dementia Rating scale (CDR) for clinical symptoms. Estimated years from symptom onset (EYO) were calculated for the SMCs by subtracting the participant’s age at which he/she first developed symptoms of progressive cognitive decline from his/her current age, and for the PMC group by subtracting the mean family age at symptom onset from his/her current age. All subjects had undergone clinical diagnosis and were aware of their mutation status. All subjects gave written informed consent according to the Declaration of Helsinki, and the study was approved by the Medical Research Ethics Committee at Xuanwu Hospital.

Additional inclusion criteria for the current study were as follows: subjects had T1, DTI and rsfMRI imaging scans from the same scanner within one scanning period, and T1 image passed quality control. Participants with any condition that might preclude completion of neuropsychological testing or MRI scanning, and with stroke, vascular disease, infarcts, hemorrhages, hydrocephalus, white matter lesions, or hyperintensities were excluded. After inclusion of subjects, further quality control was done for DTI and rsfMRI scans. The DTI quality control resulted in the exclusion of 75 scans because of incomplete coverage of the brain due to a restricted field of view, and 16 scans because of significant motion affecting the intensity of some volumes. The rsfMRI quality control resulted in the exclusion of 29 scans because of incomplete coverage of the brain due to a restricted field of view, two scans because of motion affecting the intensity of some volumes, and one scan because of missing certain number of volumes. Image exclusion steps can be found in Additional file 1: Flowchart S1.

### Image acquisition

All subjects were scanned on the same 3.0-T Siemens Skyra scanner (Germany) using a 20-channel phased array head-neck coil. Whole-brain T1-weighted three-dimensional magnetization-prepared rapid gradient echo (MPRAGE) scans (repetition time = 5000 ms, echo time = 2.98 ms, inversion time = 700 and 2500 ms, flip angle = 4°, field of view = 256 × 240 mm, matrix = 256 × 240, voxel size = 1.0 × 1.0 × 1.0 mm) were acquired. The DTI images were obtained using whole-brain 30-direction spin-echo echo planar imaging (EPI) sequence (field of view = 220 × 220 mm, matrix = 110 × 110, voxel size = 2.0 × 2.0 × 2.0 mm, 60 contiguous axial slices, repetition time = 8100 ms, echo time = 90 ms). The acquisition was performed axially with an anterior-to-posterior phase-encoding direction. Twelve reference volume (*b* = 0 s/mm^2^) and 90 diffusion volumes (*b* = 1000 s/mm^2^) with uniformly distributed diffusion directions were acquired. RsfMRI scans were collected using a gradient EPI sequence (repetition time = 2500 ms, echo time = 30 ms, flip angle = 90°, field of view = 210 × 210 mm, matrix = 70 × 70, 43 axial slices, slice thickness = 3 mm, voxel size = 3.0 × 3.0 × 3.0 mm). Participants were required to keep their eyes closed during the resting-state scanning. Imaging data were stored in DICOM format (.dcm) and converted to nifty format (.nii) using dcm2nii software for processing.

### T1 image processing and volume of gray matter ROI

T1 images were preprocessed using fslmaths and FreeSurfer software [41] (Additional file 1), which resulted in an additional exclusion of nine T1 scans. After preprocessing, six subcortical gray matter regions of interest (ROIs) including bilateral caudate, putamen, and hippocampus were obtained for each subject; four cortical gray matter ROIs were obtained, including bilateral PCC and rostral middle frontal gyrus (rMFG), which likely represents DLPFC based on a previous study [42]. The volume (absolute volume) of each ROI for each subject was calculated from the FreeSurfer software automatically. Then the relative volume of each ROI for each subject was calculated (the percentage of ROI absolute volume in intracranial volume), so as to correct the effect of difference in brain size among subjects.

### DTI processing and structural connectivity of neural circuits

DTI images were preprocessed using FSL software [43, 44] (Additional file 1), which resulted in an additional exclusion of three DTI scans. After preprocessing, the DTI data were analyzed using Probtrack (probabilistic tracking) module in FSL software [43]. Specifically, the bilateral caudate, putamen, and hippocampus were set as seed ROIs separately, and bilateral rMFG and PCC were set as waypoint masks separately. At the end, six white matter tracts (fdt paths) were obtained, including bilateral caudate-rMFG tracts, putamen-rMFG tracts, and hippocampus-PCC tracts. After obtaining the fdt paths, masks for each tract were generated with the threshold of 100. Diffusion parameters including fractional anisotropy (FA), mean diffusivity (MD), axial diffusivity (AxD), and radial diffusivity (RD) were measured using fslstats command. Fiber numbers were obtained from the waytotal output file.

### RsfMRI processing and functional connectivity of neural circuits

The data of rsfMRI were preprocessed using SPM [45] and BRANT software [46] (Additional file 1), which resulted in an additional exclusion of seven scans with excess motion (translation or rotation parameters above 1.5 mm or 1.5°). After preprocessing, a seed-based approach was performed to calculate the functional connectivity (FC) [47, 48]. Mean rsfMRI signals were extracted from each of the ROIs separately by averaging the time course signals of all voxels within the ROI. Pearson’s correlation coefficients were computed between caudate, putamen, and rMFG and between the hippocampus and PCC. The correlation coefficients (*r* values) of each pair of regions were transformed to *z* values by Fisher’s *Z* transformation process to make it in accordance with Gaussian distribution [49]. Then *z* values of each pair of regions of each individual, representing FC between two ROIs, were used for subsequent group comparisons.

### Statistical analyses

All statistical analyses were performed using SPSS 22.0. For demographic data, continuous variables were compared between groups (PMC vs. Control, and SMC vs. PMC) using the independent sample *t*-test, and categorical variables were compared between groups using the chi-square test. For imaging data including relative volume of each ROI, diffusion parameters and FC of each tract, outliers (> 2SD) were excluded first from each group. Then, data were compared among control, PMC, and SMC groups using UNIANOVA, controlling for age and sex. Then the PMC group was divided into *PS1* group, *APP* group, and *PS2* group. Demographic data were further compared in PMC between *PS1* and *APP* groups (*PS2* group was ignored due to a small sample size). *PS1* and *APP* mutation groups were compared with the control group using UNIANOVA, controlling for age and sex, to see the effect of specific gene mutation on structural and functional connectivity of the neural circuits. Then the SMC group was divided into MCI and dementia groups. Due to the limited sample size for DTI data, UNIANOVA was used to only compare ROI volume and FC among PMC (pre-MCI), MCI, and dementia groups, controlling for age and sex, to see the effect of early and late disease progression on the neural circuits. Bonferroni correction was used to correct for multiple comparison. Finally, partial correlation analyses controlling for age, sex, and education were performed for the imaging measures that showed group differences, to see the association of them with neuropsychological and clinical measures including MMSE total score, MoCA total score, CDR global score, and EYO.

## Results

### Subject characteristics

Detailed demographic information are presented in Table 1. The PMC group was younger than the control group [*t*(140) = − 3.224, *P* = 0.002] and SMC group [*t*(65.2) = − 4.720, *P* < 0.001]. The SMC group had a higher male to female ratio than the PMC group (*X*^*2*^ = 1.595, *P* = 0.038). The SMC group had higher EYO than the PMC group [*t*(45.4) = − 7.253, *P* < 0.001]. The three groups were similar in education level and proportion of *APOEε4*. SMC and PMC had similar proportion of the three known gene mutations. The SMC group had lower MMSE [*t*(29.0) = − 7.973, *P* < 0.001] and MoCA [*t*(32.9) = − 9.392, *P* < 0.001] scores than the PMC group. Furthermore, in the PMC group, the *PS1* and *APP* mutation groups were not different in age (*PS1*: 34.0 ± 14.7 years, *APP*: 35.7 ± 15.4 years) or EYO (*PS1*: − 11.1 ± 13.4 years, *APP*: − 13.2 ± 13.7 years).

**Table 1:** Subject demographics, neuropsychological, and clinical data

	Control (*n* = 102)	PMC (*n* = 40)	SMC (*n* = 30)	*P* value PMC vs. Control	*P* value SMC vs. PMC
Age (years)	44.4 (12.6)	36.3 (15.7)	50.5 (9.4)	0.002**	< 0.001***
Sex (male/female)	37/65	14/26	18/12	0.887	0.038^#^
Education (years)	11.0 (5.3)	12.1 (4.1)	10.5 (4.2)	0.249	0.118
EYO (years)	N/A	−11.6 (13.2)	4.1 (3.1)		< 0.001***
MMSE	29.0 (1.6)	28.8 (1.8)	16.5 (8.0)	0.498	< 0.001***
MoCA	27.1 (2.3)	27.1 (3.0)	12.7 (7.7)	0.872	< 0.001***
CDR	N/A	N/A	1.3 (0.8)		
Subject numbers					
*APP*/*PS1*/*PS2*	N/A	17/21/2	9/18/3		0.474
*APOE*ε4 (Y/N)	26/76	8/32	10/20	0.490	0.207
T1 MRI after PP	95	40	28		
DTI after QC+PP	45	24	9		
rsfMRI after QC+PP	84	30	19		

For continuous variables, data are shown in mean (SD), and the independent sample *t*-test was used for group comparison; for categorical variables, the chi-square test was used for group comparison. Bonferroni correction was used to correct for multiple comparison. ^#^0.025 < *P* < 0.05, **0.001 < *P* < 0.01, ***P < 0.001

*Y/N* the number of subjects carrying (Y) or not carrying (N) *APOE*ε4, *PP* preprocessing, *QC* quality control, *PMC* presymptomatic mutation carriers, *SMC* symptomatic mutation carriers, *EYO* estimated years from symptom onset, *MMSE* Mini-Mental State Examination, *MoCA* Montreal Cognitive Assessment, *CDR* Clinical Dementia Rating scale

### Overall group comparison of the structure and function of neural circuits

**Fig. 1:**
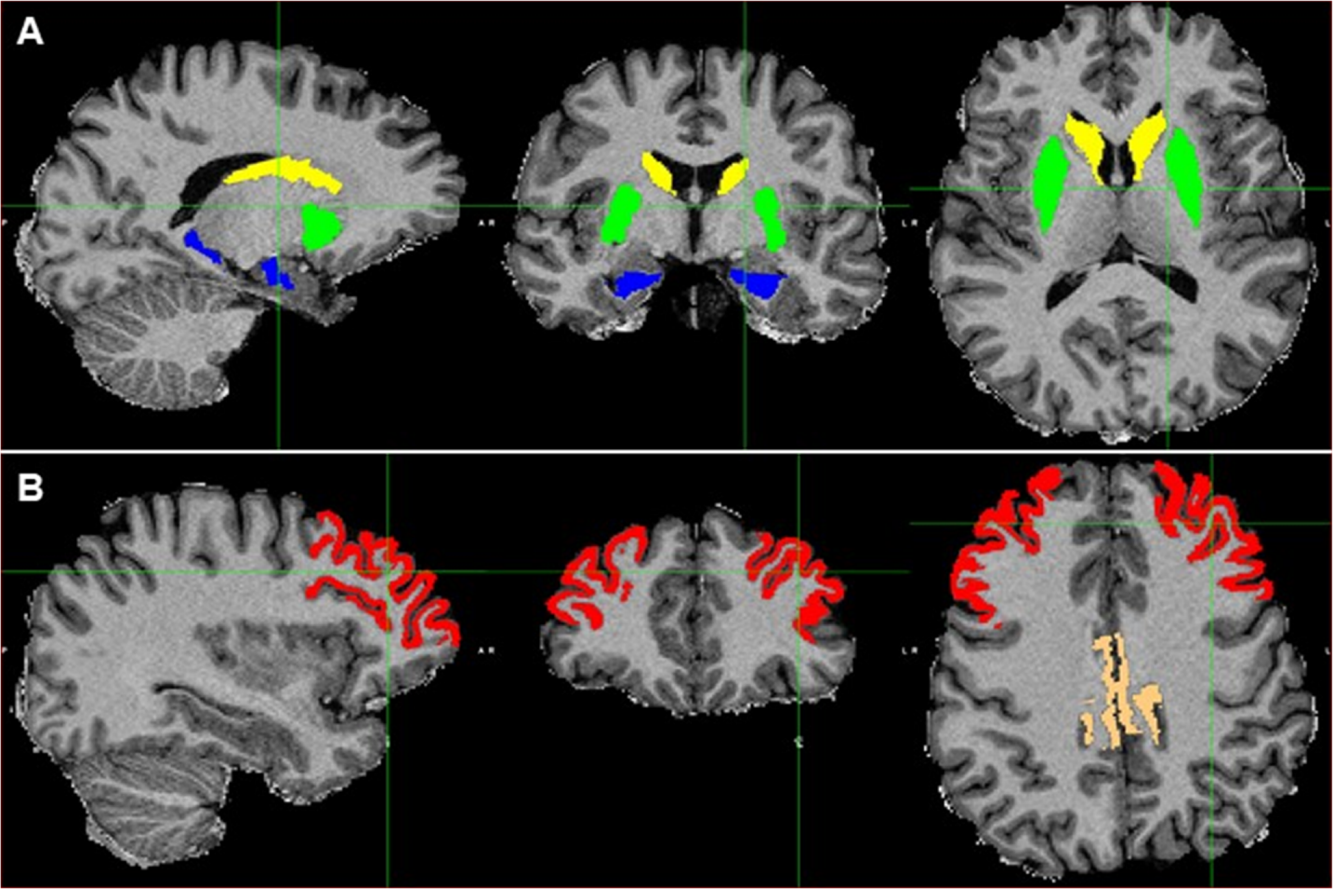
The ROIs of a representative subject in structural space. The subcortical ROIs (**a**) include the bilateral caudate (yellow), putamen (green), and hippocampus (blue); the cortical ROIs (**b**) include rMFG (red) and PCC (brown)

**Fig. 2:**
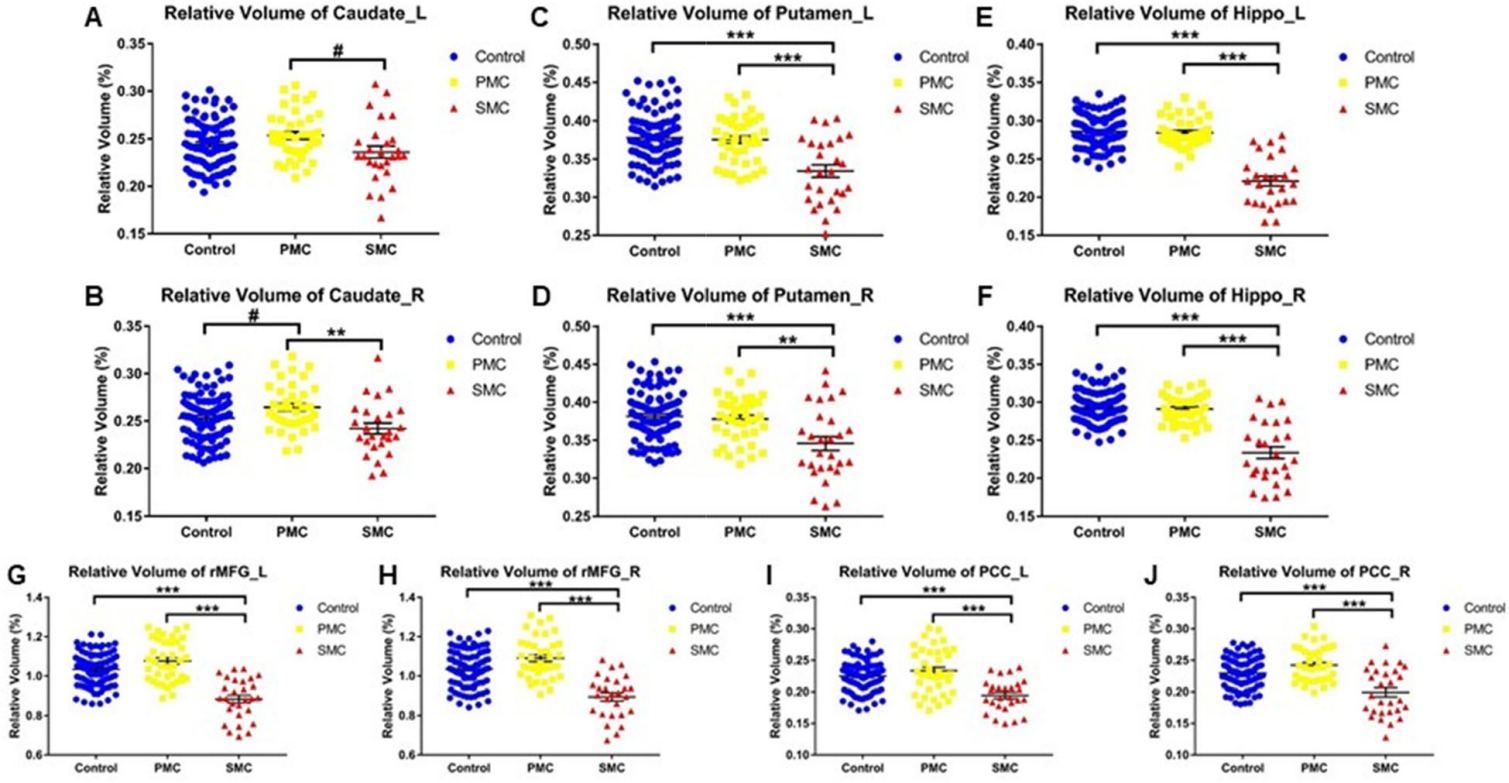
Overall group comparison of the ROI volume. **a**) left caudate, **b**) right caudate, **c**) left putamen, **d**) right putamen, **e**) left hippocampus, **f**) right hippocampus, **g**) left rMFG, **h**) right rMFG, **i**) left PCC, **j**) right PCC. Relative volume of each subject is calculated as the percentage of absolute volume in intracranial volume. The bars indicate mean (SD). rMFG: rostrol middle frontal gyrus; PCC: posterior cingulate cortex; Hippo: hippocampus; L: left; R: right. ^#^0.0167 < *P* < 0.05, *0.01 < *P* < 0.0167, **0.001 < *P* < 0.01, ***P < 0.001

**Fig. 3:**
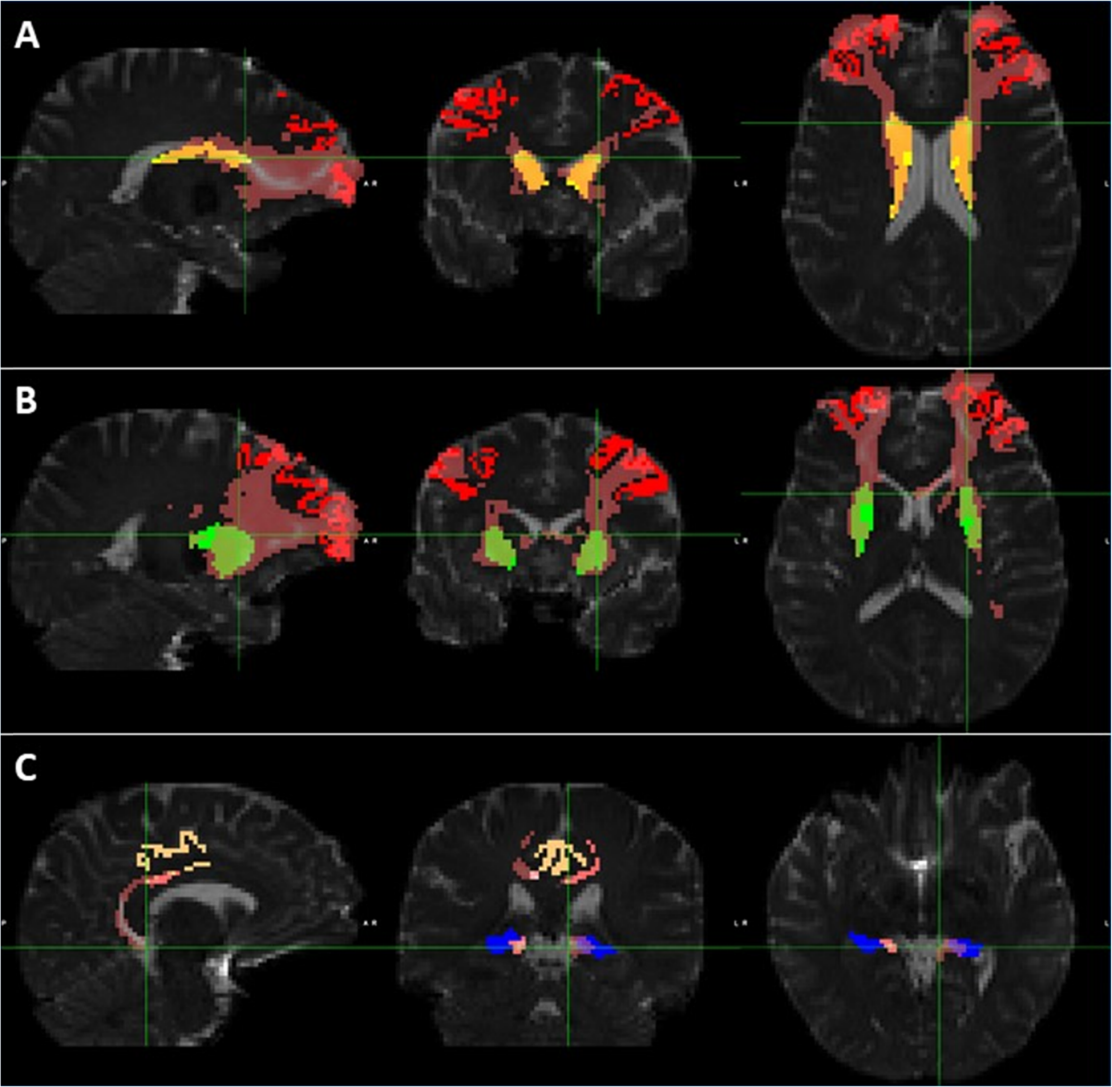
The ROIs and tracts of a representative subject in diffusion space. The tracts (pink) include the bilateral caudate-rMFG (**a**), putamen-rMFG (**b**), and hippocampus-PCC (**c**). The ROIs are in the same color with those in Fig. 1

**Fig. 4:**
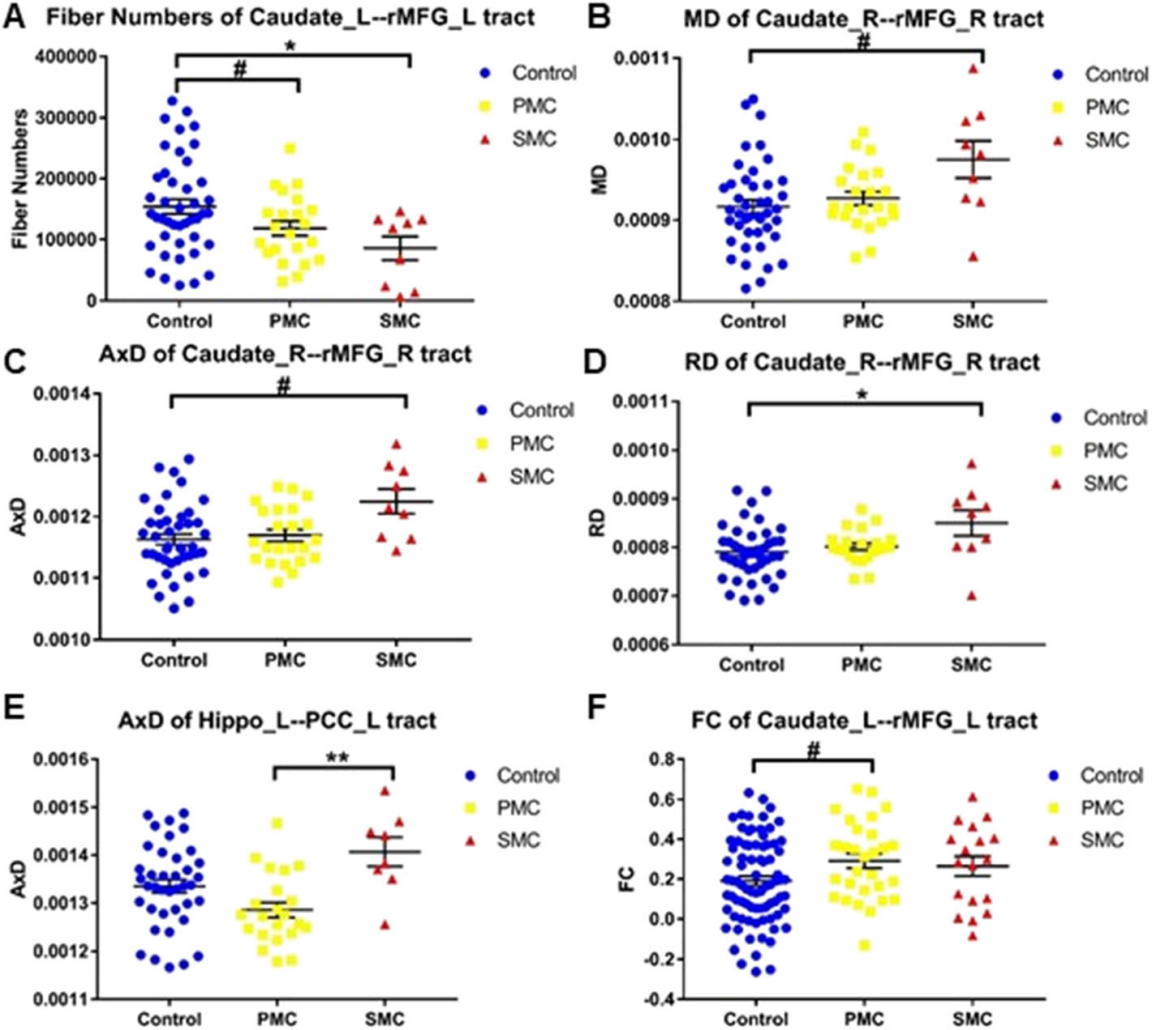
Overall group comparison of the structural and functional connectivity of neural circuits. **a**) fiber numbers of left caudate-rMFG tract, **b**) MD of right caudate-rMFG tract, **c**) AxD of right caudate-rMFG tract, **d**) RD of right caudate-rMFG tract, **e**) AxD of left hippo-PCC tract, **f**) FC of left caudate-rMFG tract. MD: mean diffusivity; AxD: axial diffusivity; RD: radial diffusivity; FC: functional connectivity. The bars indicate mean (SD). ^#^0.0167 < *P* < 0.05, *0.01 < *P* < 0.0167, **0.001 < *P* < 0.01

Figure 1 shows the ROIs of a representative subject in structural space. UNIANOVA showed that there were group differences in the volume of all the ROIs (Fig. 2, *P*s < 0.05) except for the left caudate which showed a marginal difference (*F* = 2.604, *P* = 0.077). Compared with the control group, PMC showed a trend level volume increase in the right caudate (Fig. 2b, *P* = 0.039); SMC showed volume reduction in the bilateral putamen, hippocampus, rMFG, and PCC (*P*s < 0.0167). Compared with PMC, SMC showed volume reduction in all ROIs (*P*s < 0.0167) except for the left caudate which showed a trend level difference (*P* = 0.029). Figure 3 shows the white matter tracts of a representative subject in diffusion space. UNIANOVA among control, PMC, and SMC showed that there were group differences in the fiber numbers (*F* = 4.456, *P* = 0.015) of left caudate-rMFG tract; MD (*F* = 3.510, *P* = 0.035), AxD (*F* = 3.310, *P* = 0.042) and RD (*F* = 3.930, *P* = 0.024) of right caudate-rMFG tract; and AxD (*F* = 4.574, *P* = 0.014) of left hippocampus-PCC tract (Fig. 4). Compared with the control group, PMC showed trend level reduced fiber numbers (*P* = 0.044) of the left caudate-rMFG tract; SMC showed significant reduced fiber numbers (*P* < 0.0167) of the left caudate-rMFG tract, trend level increased MD (*P* = 0.020) and AxD (*P* = 0.022), and significant increased RD (*P* < 0.0167) of the right caudate-rMFG tract. Compared with PMC, SMC showed significantly increased AxD (*P* < 0.0167) of the left hippocampus-PCC tract. Medial and lateral views of bilateral ROIs in standard space are shown in Additional file 1: Figure S1. UNIANOVA among control, PMC, and SMC showed a trend level group difference in FC of the left caudate-rMFG tract (*F* = 2.923, *P* = 0.057). Compared with the control group, PMC showed a trend level increased FC (Fig. 4f, *P* = 0.024).

### Effects of gene mutation on the structure and function of neural circuits

**Fig. 5:**
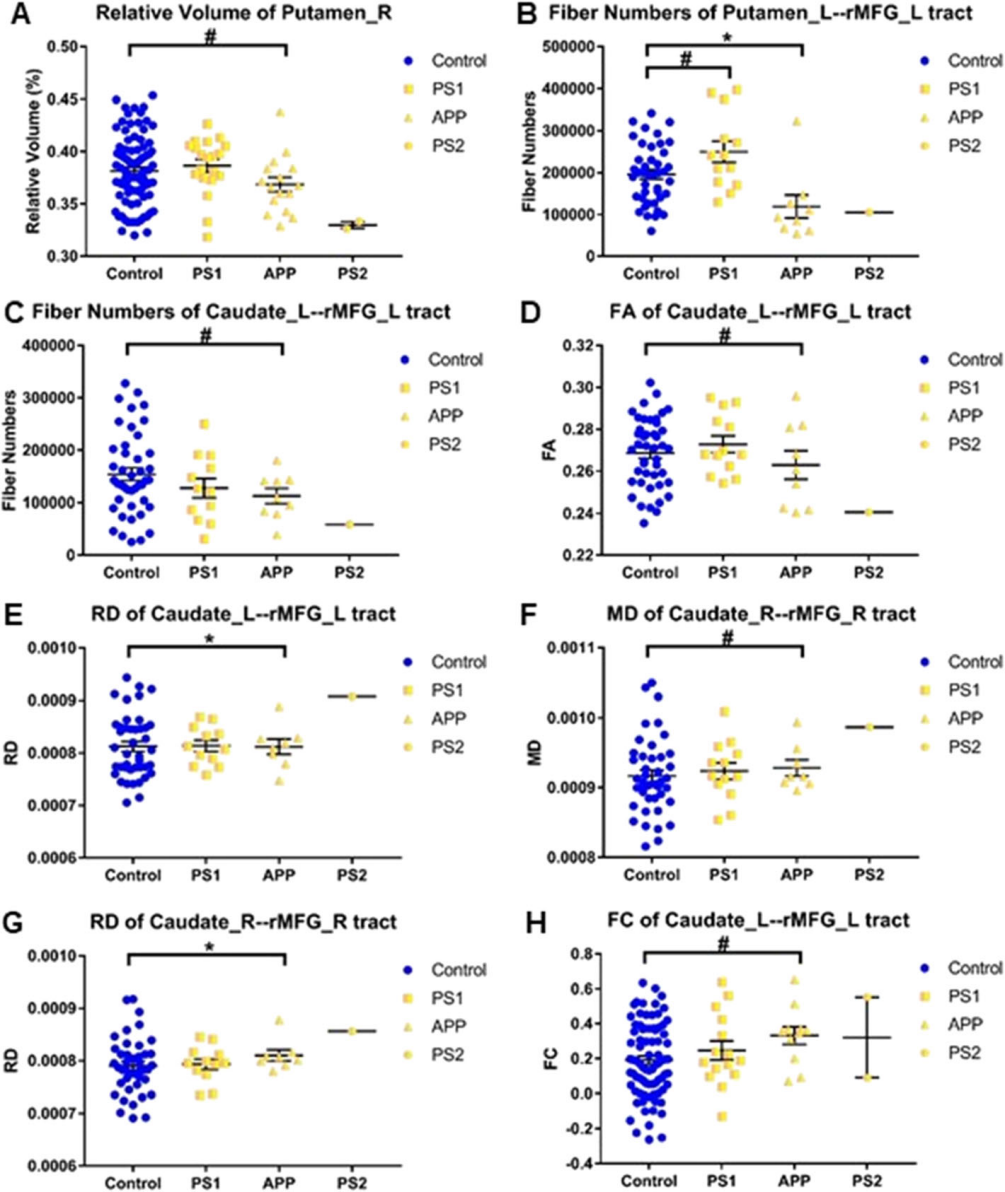
Effects of gene mutation on the imaging measures of neural circuits. **a**) relative volume of right putamen, **b**) fiber numbers of left putamen-rMFG tract, **c**) fiber numbers of left caudate-rMFG tract, **d**) FA of left caudate-rMFG tract, **e**) RD of left caudate-rMFG tract, **f**) MD of right caudate-rMFG tract, **g**) RD of right caudate-rMFG tract, **h**) FC of left caudate-rMFG tract. *PS1* and *APP* mutation subjects without symptoms were compared with the control group, respectively. *PS2* mutation subjects were listed for reference though not compared with the control group due to the small sample size. The bars indicate mean (SD). ^#^ 0.025 < *P* < 0.05, * 0.01 < *P* < 0.025

In PMC, subjects were divided into *PS1*, *APP*, and *PS2* mutation groups. Due to the small sample size of *PS2* group, we only compared the *PS1* and *APP* groups with the control group. In the *APP* mutation group, there was a trend level decreased volume in the right putamen (Fig. 5a, *F* = 3.972, *P* = 0.049); significantly reduced fiber numbers of the left putamen-rMFG tract (Fig. 5b, *F* = 5.630, *P* < 0.025); trend level reduced fiber numbers (Fig. 5c, *F* = 4.360, *P* = 0.042), reduced FA (Fig. 5d, *F* = 4.134, *P* = 0.047), and significantly increased RD (Fig. 5e, *F* = 7.175, *P* < 0.025) of the left caudate-rMFG tract; trend level increased MD (Fig. 5f, *F* = 4.189, *P* = 0.046) and significantly increased RD (Fig. 5g, *F* = 6.488, *P* < 0.025) of the right caudate-rMFG tract; and trend level increased FC of the left caudate-rMFG tract (Fig. 5h, *F* = 4.150, *P* = 0.045). The *PS1* mutation group showed trend level increased fiber numbers of the left putamen-rMFG tract (Fig. 5b, *F* = 4.789, *P* = 0.033).

### Effects of disease progression on the structure and function of neural circuits

**Fig. 6:**
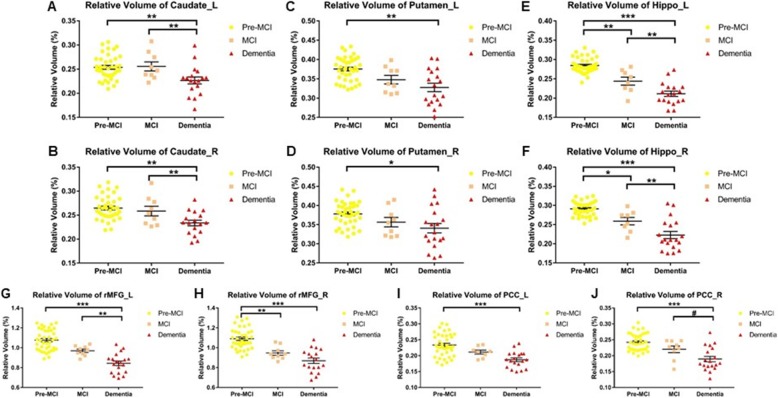
Comparison of ROI volume among different stages of mutation carriers. **a**) left caudate, **b**) right caudate, **c**) left putamen, **d**) right putamen, **e**) left hippocampus, **f**) right hippocampus, **g**) left rMFG, **h**) right rMFG, **i**) left PCC, **j**) right PCC. Pre-MCI represents presymptomatic stage (original PMC group). The bars indicate mean (SD). ^#^ 0.0167 < *P* < 0.05, * 0.01 < *P* < 0.0167, ** 0.001 < *P* < 0.01, ***P < 0.001

In mutation carriers, UNIANOVA showed volume reduction of bilateral hippocampus (Fig. 6e, f, *P* = 0.002 for left, *P* = 0.016 for right) and right rMFG (Fig. 6h, *P* = 0.003) in MCI stage; and volume reduction of all the ROIs in dementia stage (Fig. 6, *P*s < 0.0167) as compared with pre-MCI stage. Furthermore, as compared with MCI stage, bilateral caudate (Fig. 6a, b, *P* = 0.008 for left, *P* = 0.009 for right), bilateral hippocampus (Fig. 6e, f, *P* = 0.001 for left, *P* = 0.002 for right), and left rMFG (Fig. 6g, *P* = 0.001) showed volume reduction in dementia stage. RsfMRI data did not show group difference in FC of any tract (figure not shown).

### Neuropsychological and clinical correlations of neural circuits

Additional file 1: Figure S2 shows the results of neuropsychological and clinical correlations of the imaging measures of neural circuits that showed overall group differences. In SMC, AxD of the right caudate-rMFG tract correlated significantly negatively with MMSE (*r* = − 0.939, *P* = 0.018) and MoCA total score (*r* = − 0.905, *P* = 0.035), and significantly positively with CDR global score (*r* = 0.995, *P* = 0.005); FC of the left caudate-rMFG tract correlated significantly positively with MMSE total score (*r* = − 0.590, *P* = 0.034). In addition, there were significant correlations of relative volume in all the ROIs with MMSE, MoCA, and CDR global score (Additional file 1: Figure S3, S4 and S5), but not with EYO. Combining SMC and PMC, AxD of the left hippocampus-PCC tract correlated significantly negatively with MMSE (*r* = − 0.558, *P* = 0.003) and MoCA total score (*r* = − 0.531, *P* = 0.005). In PMC, RD of right caudate-rMFG tract correlated significantly positively with MMSE total score in *PS1* carriers (*r* = 0.749, *P* = 0.020) but not in *APP* carriers (*r* = 0.565, *P* > 0.05); relative volume of right putamen correlated significantly positively with MMSE total score in *PS1* carriers (*r* = 0.511, *P* = 0.036) but not in *APP* carriers (*r* = 0.198, *P* > 0.05). In the control group, none of the imaging measures showed significant correlation with neuropsychological or clinical measures (figure not shown).

## Discussion

To the best of our knowledge, this is the first study looking at the effects of different gene mutations and disease progression on the specific striatum- and hippocampus-related neural circuits in FAD. The major findings were that *APP* and *PS1* gene mutations affected the structure and function of the frontostriatal pathway in a different manner in presymptomatic stage, where *APP* primarily impaired the structural connectivity; disease progression especially affected the structure of hippocampus-PCC pathway; and structural connectivity of both frontostriatal and hippocampus-PCC circuits were associated with general cognitive function in FAD. Our findings may deepen our understanding of the circuit-based imaging biomarkers of Alzheimer’s disease and pave the way for early identification, prediction of prognosis, and development of gene- or circuit-targeted treatment and prevention.

For the overall group comparison, our results primarily indicate increased volume, reduced fiber numbers, and elevated function of the caudate-rMFG tract at the presymptomatic stage, while later impairment of the structure of both caudate-rMFG and hippocampus-PCC tracts. Regarding volume changes of the caudate and hippocampus in PMC of FAD, the results of previous studies were inconsistent. While some reported volume increase of the caudate [23], some reported volume reduction of the caudate [19, 20] and hippocampus [16, 21, 50], and some reported no volume change of the caudate [51] or hippocampus [20, 23]. Such discrepancy might be due to the differences in subjects’ demographics (such as EYO, mutations, and age), small sample sizes, or different image segmentation and statistical methods [39]. Caudate volume, like some key cortical regions, might follow an inverted U-shape nonlinear pattern over time from presymptomatic to symptomatic stages, as evidenced by longitudinal imaging studies [15, 51], which could be explained by initial reactive neuronal hypertrophy, neuroinflammation, and/or amyloid accumulation followed by neurodegeneration. Regarding structural connectivity changes, previous DTI studies showed a similar nonlinear pattern in tracts containing caudate or hippocampus, including increased FA and decreased diffusivity in PMC, while increased diffusivity in SMC [20, 23, 52], which might reflect earlier pathological changes such as inflammation, microglial activation/accumulation, and swelling of neurons and glia, while later impairment of white matter integrity of cellular structures [20]. Our results in both the caudate-rMFG and hippocampus-PCC tracts are consistent with such pattern though not significant in PMC. On the other hand, reduced fiber numbers of the caudate-rMFG tract in our study might reflect loss of axons or myelination [53], indicating white matter tract degeneration [54]. Regarding functional changes, our results in the caudate-rMFG tract were also consistent with an inverted U-shape nonlinear pattern though not significant in SMC, probably due to a small sample size or early in the disease progression (short EYO) in SMC. Increased connectivity may indicate high processing burden and/or noisy inefficient synaptic communication [55], as highly connected regions are particularly vulnerable to Aβ deposition because of their increased synaptic activity, according to the “nodal stress” hypothesis [56–58].

For the effect of gene mutation on the structure and function of the representative neural circuits, our results indicate that *APP* mutation has early deleterious effect on the structural integrity of frontostriatal neural circuits, while *PS1* mutation has little or even compensatory effect on it. The detrimental effects of *APP* mutation on frontostriatal pathway include loss of axons or myelination, reduction in white matter integrity and disruption of white matter microstructures as measured by fiber numbers [53, 54], FA [59], and MD [52], respectively. In addition, AxD and RD, the principal direction and perpendicular direction of the diffusion ellipsoid, have been shown to assess axonal integrity and myelin integrity, respectively [60]. Our findings indicate that *APP* mutation primarily affects myelin integrity while preserving axonal integrity. Furthermore, *APP* mutation is the primary contributor to the increased FC in the caudate-rMFG tract, which might be due to a compensatory mechanism that prevents cognitive decline, or reflect the early pathological changes. On the contrary, the little or compensatory effect of *PS1* mutation on the frontostriatal circuits is increased axons or myelination in putamen-rMFG tract. Neuronal hypertrophy has been shown in cognitive normal subjects with AD pathologies, which might be a probable reaction to Aβ [61]. There is evidence showing different levels of damage to the right DLPFC-right caudate-left thalamus-right DLPFC circuit in different MCI groups (reverted to normal, stable, or progressed to dementia), which indicates that FC of the frontostriatal circuit might be a potential biomarker for early detection of Alzheimer’s disease [62]. Our findings of the different effects of *APP* and *PS1* mutation on this circuit expanded our understanding of this potential biomarker. Both *APP* and *PS1* mutation can cause direct elevation of the Aβ level via alterations of their encoding proteins and, as a result, affect synaptic plasticity [40, 63]. They also affect other functions of their encoding proteins, such as axonal transport, lysosome function, and autophagy [64–66], all of which can affect the properties of neural circuits. The lysosomal-autophagy defects are dependent on *APP* protein and not independent phenotypes due to either *APP* or *PS1* mutation. Furthermore, *PS1* mutant neurons do not have early endosome defects, in contrast with *APP* mutant neurons [65]. These evidence may partially explain the different effects of *APP* and *PS1* mutation on the neural circuits in our study.

For the effect of disease progression on the representative neural circuits, our results indicate that disease progression has deleterious effect on the structure of the frontostriatal and hippocampus-PCC circuits (especially the latter), while no effect on the function of them. Imaging studies in early symptomatic stage of FAD found cortical thinning of prefrontal cortices [51] and atrophy of the hippocampus [67], which were consistent with our findings of rMFG and hippocampal atrophy in MCI stage. Imaging studies in FAD have also shown increased diffusivity of the hippocampus, cingulum, and hippocampus-cingulum tract from PMC to SMC [20, 23, 52]. A longitudinal imaging study found that increased diffusivity of the hippocampus-PCC tract predicted tau accumulation in the downstream-connected PCC and memory decline in amyloid-positive subjects [68], which further supported that the structural connectivity of the hippocampus-PCC circuit is a potential biomarker for disease progression and in line with the amyloid and tau hypothesis. Our findings of increased AxD while no change in RD of the hippocampus-PCC tract indicate that disease progression primarily affects axonal integrity while preserving myelin integrity. There is evidence of increased diffusivity (especially AxD) of cingulum angular bundles connecting to the hippocampus in individuals with MCI and sporadic Alzheimer’s disease even after controlling for hippocampal volume [69], suggesting that axonal damage of the hippocampus-PCC circuit additionally contributes to Alzheimer’s disease progression. Regarding functional changes, PCC is a key region of the posterior default mode network (DMN), in which high connectivity with ventral DMN including the hippocampus region was associated with amyloid accumulation and symptom severity in sporadic Alzheimer’s disease [55]. One study showed disrupted FC between the hippocampus and PCC in the early dementia stage, while not in the MCI stage [70]. Another study in *PS1* mutation carriers showed decreased FC between the hippocampus and PCC in PMC, but no change in SMC [71]. Such discrepancy might be due to different population, age range, and stages in the disease spectrum. Our result of no change in the FC of the hippocampus-PCC tract with disease progression might indicate a nonlinear pattern or that the subjects were in relatively early stage of the disease progression.

For the neuropsychological and clinical correlations of the representative neural circuits, our findings suggest that all the key nodes and structural connectivity (especially axonal integrity) of both frontostrial and hippocampus-PCC circuits are involved in general cognitive function and disease severity in FAD, and the presymptomatic involvement of the frontostriatal circuit structure in general cognitive function is disrupted in *APP* mutation carriers. Frontostriatal circuit functional connectivity is further involved in general cognitive function. Functional imaging studies have found that frontostriatal regions are involved in executive function and working memory [28–33], and hippocampus-PCC regions are involved in episodic memory [34, 35, 72]. Our findings indicate that not only the function, but also the structure (axonal integrity) of these circuits is involved in general cognitive function and disease severity in FAD.

**Fig. 7:**
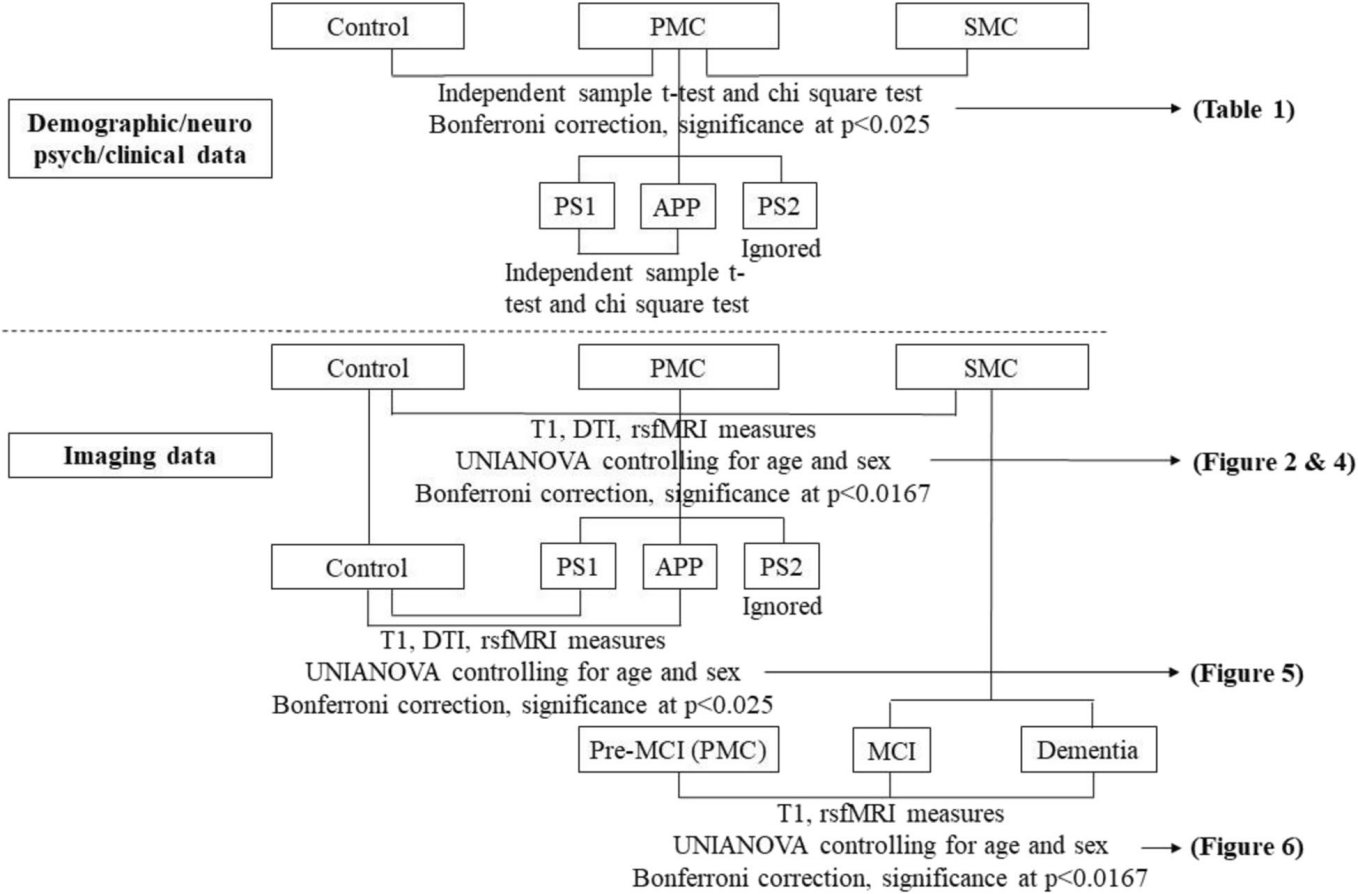
Group comparisons done

This study has several limitations. First, the sample size for DTI data was relatively small, which hindered our exploration of the effect of disease progression on the neural circuits; second, the neuropsychological and clinical evaluations tested general cognition, and thus were not specific to each cognitive function domain such as episodic memory or executive function, which might be directly associated with the neural circuits in our study; third, Bonferroni correction was used for group comparisons but not for cognitive/clinical correlations, raising the possibility of a type 1 error, and the trend level results should be preliminary and exploratory (Fig. 7). However, there are fewer studies investigating white matter neural circuits than gray matter in FAD; thus, it remains helpful to search for possible useful circuit biomarkers that future studies can replicate; fourth, the DTI measurements especially FA and MD are nonspecific and only represent the general white matter integrity, though previous studies have linked early increased FA and low MD to inflammation, microglial activation/accumulation, and swelling of neurons and glia [20]. Further histology studies are needed to specify the nature of these measurements; fifth, cardiac or respiration rates were not recorded during the scanning. Although the preprocessing steps of rsfMRI can reduce the effect of them to some extent, they may still have an influence on the FC results as reported by a study [73].

## Conclusions

*APP* and *PS1* gene mutations affect the frontostriatal neural circuit in a different manner, where *APP* primarily impairs the myelin integrity. The structure (especially axonal integrity) of the hippocampus-PCC circuit is a potential biomarker for disease progression in FAD. The structural connectivity of both frontostriatal and hippocampus-PCC circuits are associated with general cognitive function. Future studies will use a longitudinal design to evaluate the gene-specific effect and disease progression on the structure and function of the representative neural circuits, and to further link circuit imaging biomarkers with cognitive function domains.

## Supplementary information

Additional file 1 **:** Flowchart S1. Image exclusion steps. **Figure S1.** The ROIs of a representative subject in standard space. **Figure S2.** Neuropsychological and clinical correlations of the structural and functional connectivity of neural circuits. **Figure S3.** Correlations of the ROI volume with MMSE. **Figure S4.** Correlations of the ROI volume with MoCA. **Figure S5.** Correlations of the ROI volume with CDR.

## Data Availability

Both raw and processed data that support the findings of the current study will be made available upon request to the corresponding author and the CFAN committee in order to ensure that the privacy of the CFAN participants is protected.

## Abbreviations

ADAD: Autosomal dominant Alzheimer’s disease
*APOE*: Apolipoprotein E
*APP*: Amyloid precursor protein
AxD: Axial diffusivity
CDR: Clinical Dementia Rating scale
DTI: Diffusion tensor imaging
EYO: Estimated years from symptom onset
FA: Fractional anisotropy
FAD: Familial Alzheimer’s disease
FC: Functional connectivity
MD: Mean diffusivity
MMSE: Mini-Mental State Examination
MoCA: Montreal Cognitive Assessment
PCC: Posterior cingulate cortex
PMC: Presymptomatic mutation carrier
*PS1*: Presenilin 1
*PS2*: Presenilin 2
RD: Radial diffusivity
rMFG: Rostral middle frontal gyrus
ROI: Region of interest
rsfMRI: Resting-state functional MRI
SMC: Symptomatic mutation carrier
