# Supplementary material for: Effects of gene mutation and disease progression on representative neural circuits in familial Alzheimer’s disease
Source: Alzheimers Res Ther. 2020 Jan 14;12:14. doi: 10.1186/s13195-019-0572-2 (PMC6961388; doi:10.1186/s13195-019-0572-2)
Supplement: Supplementary file 1 — : Flowchart S1. Image exclusion steps. Figure S1. The ROIs of a representative subject in standard space. Figure S2. Neuropsychological and clinical correlations of the structural and functional connectivity of neural circuits. Figure S3. Correlations of the ROI volume with MMSE. Figure S4. Correlations of the ROI volume with MoCA. Figure S5. Correlations of the ROI volume with CDR. [file 13195_2019_572_MOESM1_ESM.docx]

***Additional file 1***

**Effects of gene mutation and disease progression on representative neural circuits in familial Alzheimer’s disease**

Meina Quan^1-4^, Tan Zhao^1-4^, Yi Tang^1-4^, Ping Luo^5^, Wei Wang^1-4^, Qi Qin^1-4^, Tingting Li^1-4^, Qigeng Wang^1-4^, Jiliang Fang^5^, Jianping Jia^1-4^*

^1^ Innovation Center for Neurological Disorders and Department of Neurology, Xuanwu Hospital, Capital Medical University, National Clinical Research Center for Geriatric Diseases, Beijing, PR China

^2^ Beijing Key Laboratory of Geriatric Cognitive Disorders, Beijing, PR China

^3^ Clinical Center for Neurodegenerative Disease and Memory Impairment, Capital Medical University, Beijing, PR China

^4^ Center of Alzheimer's Disease, Beijing Institute for Brain Disorders, Beijing, PR China

^5^ Guang'anmen Hospital, China Academy of Chinese Medical Sciences, Beijing, China

**Methods:**

**Image preprocessing for T1**

T1 images for each subject were preprocessed using fslmaths command with a threshold of 80 for reduction of the background noise, and using the FreeSurfer software package version 5.3.0 (http://surfer.nmr.mgh.harvard.edu) [1][, for brain-extraction and segmentation into cortical and subcortical gray and white matter regions.](#_ENREF_10)

**Image preprocessing for DTI**

DTI images were preprocessed using FSL software (https://fsl.fmrib.ox.ac.uk/fsl/fslwiki) FDT toolbox, including modules of brain extraction (BET) for brain mask generation with a fractional intensity threshold of 0.2, Eddy correct for correction of eddy current distortions, DTIFIT for head motion correction and reconstruction of diffusion tensors, and Bedpostx for local modelling of diffusion parameters [2]. Individual brain images were visually inspected for signal dropout, artifacts, and other distortions. Then brain-extracted DTI images were registered with betted and non-betted T1 image of the same subject and Montreal Neurological Institute (MNI) standard space image using Registration module. After getting the transformation matrix, the ROIs of each subject were reoriented from FreeSurfer space to structural space, and then were registered to the diffusion space using FSL’s linear image registration tool (FLIRT), with nearest neighbor interpolation [3], and to the MNI standard space using FSL’s nonlinear registration tool (FNIRT), for the subsequent use in rsfMRI functional connectivity analysis.

**Image preprocessing for rsfMRI**

The data of rsfMRI were preprocessed by SPM12 (http://www.fil.ion.ucl.ac.uk/spm) [4] and BRANT (http://brant.brainnetome.org) [5]. For each participant, the following steps were implemented, including (i) slice timing correction; (ii) head motion correction (each volume was realigned to the first volume); (iii) co-registration of segmented T1 image (grey matter GM, white matter WM, and cerebrospinal fluid CSF) with the mean rsfMRI image; (iv) spatial normalization of the mean rsfMRI image to the MNI standard space; (v) spatial smoothing using Gaussian kernel with full-width at half maximum of 6mm; (vi) linear trend, mean time series extracted from tissue masks (WM, CSF and global signal) and six head motion parameters were regressed out through a multiple linear regression analysis; (vii) temporal filtering using a 0.01-0.08 Hz band-pass filter.

**Flowchart S1: Image exclusion steps.**

**
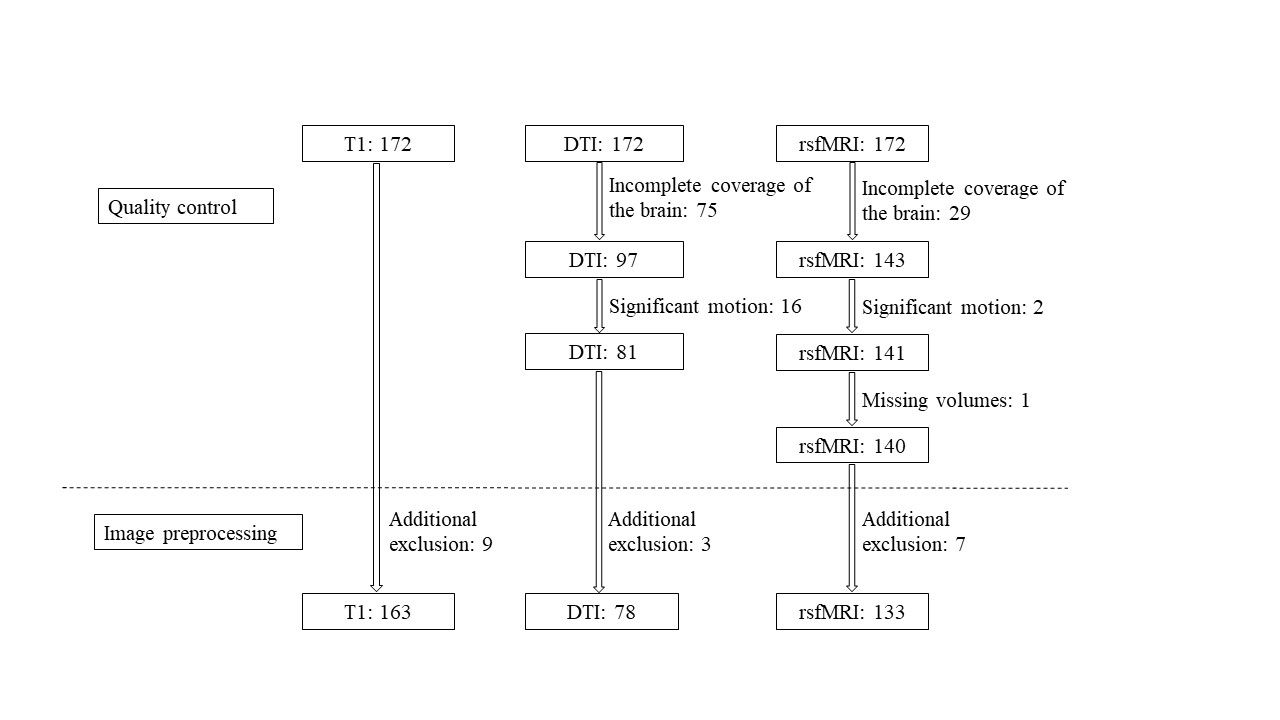
**

**Figure S1. The ROIs of a representative subject in standard space.**


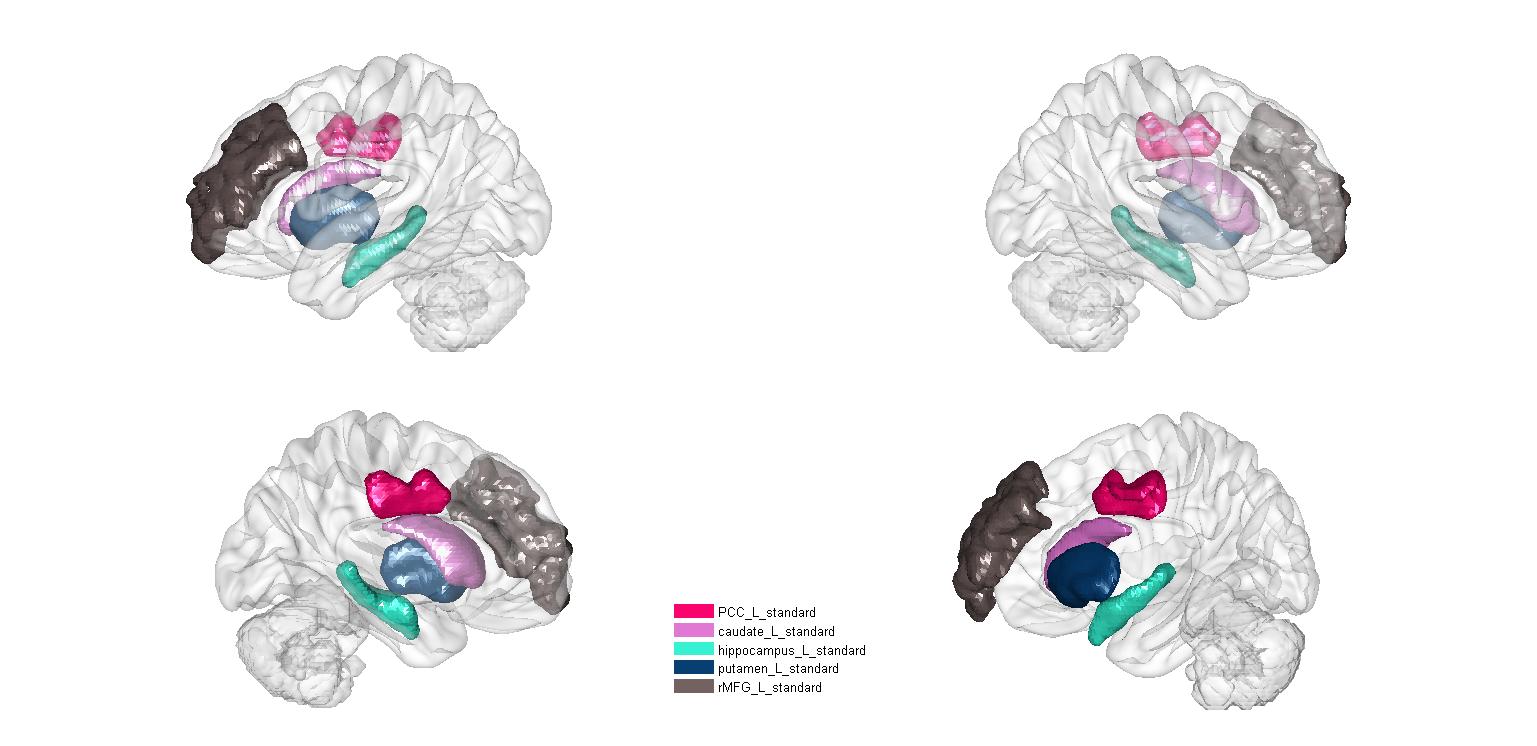

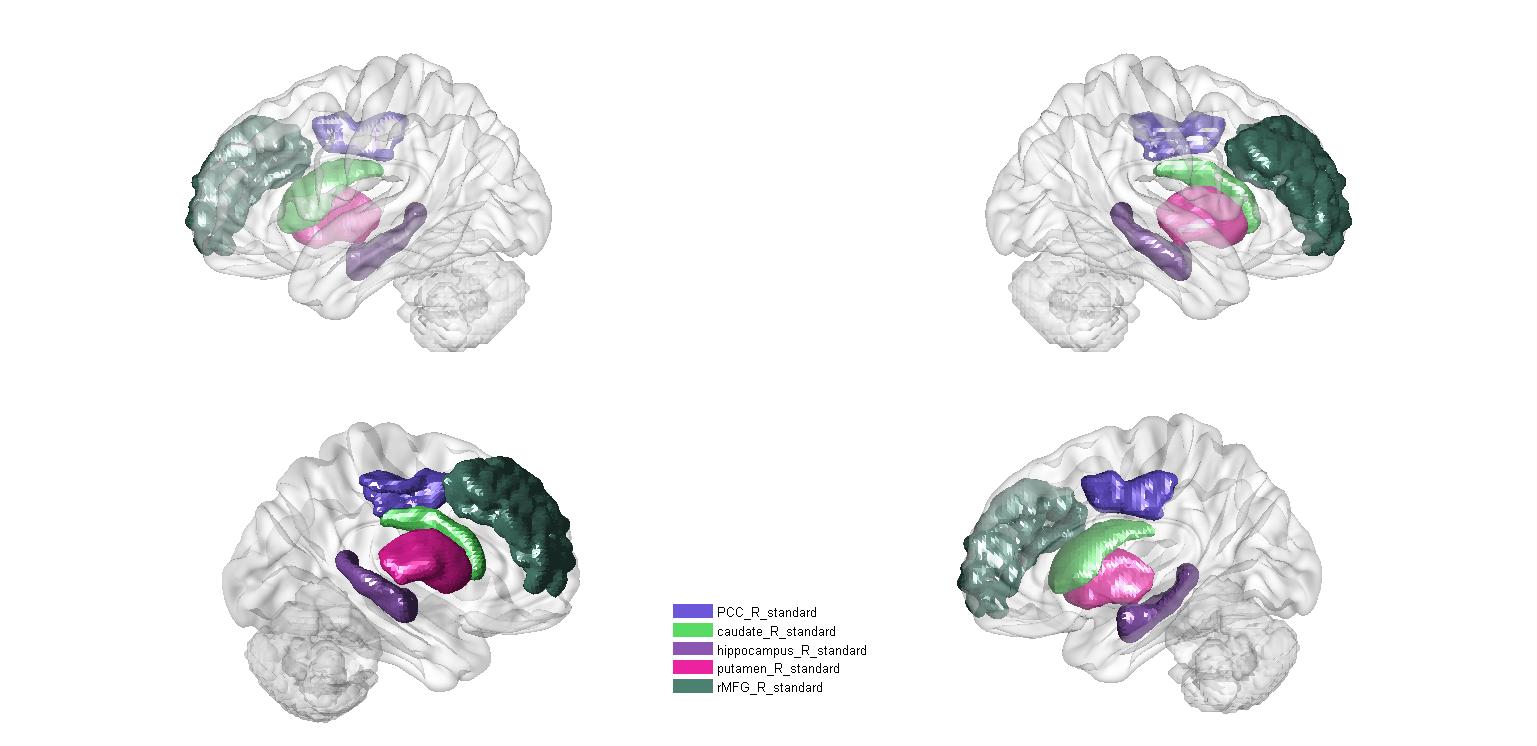

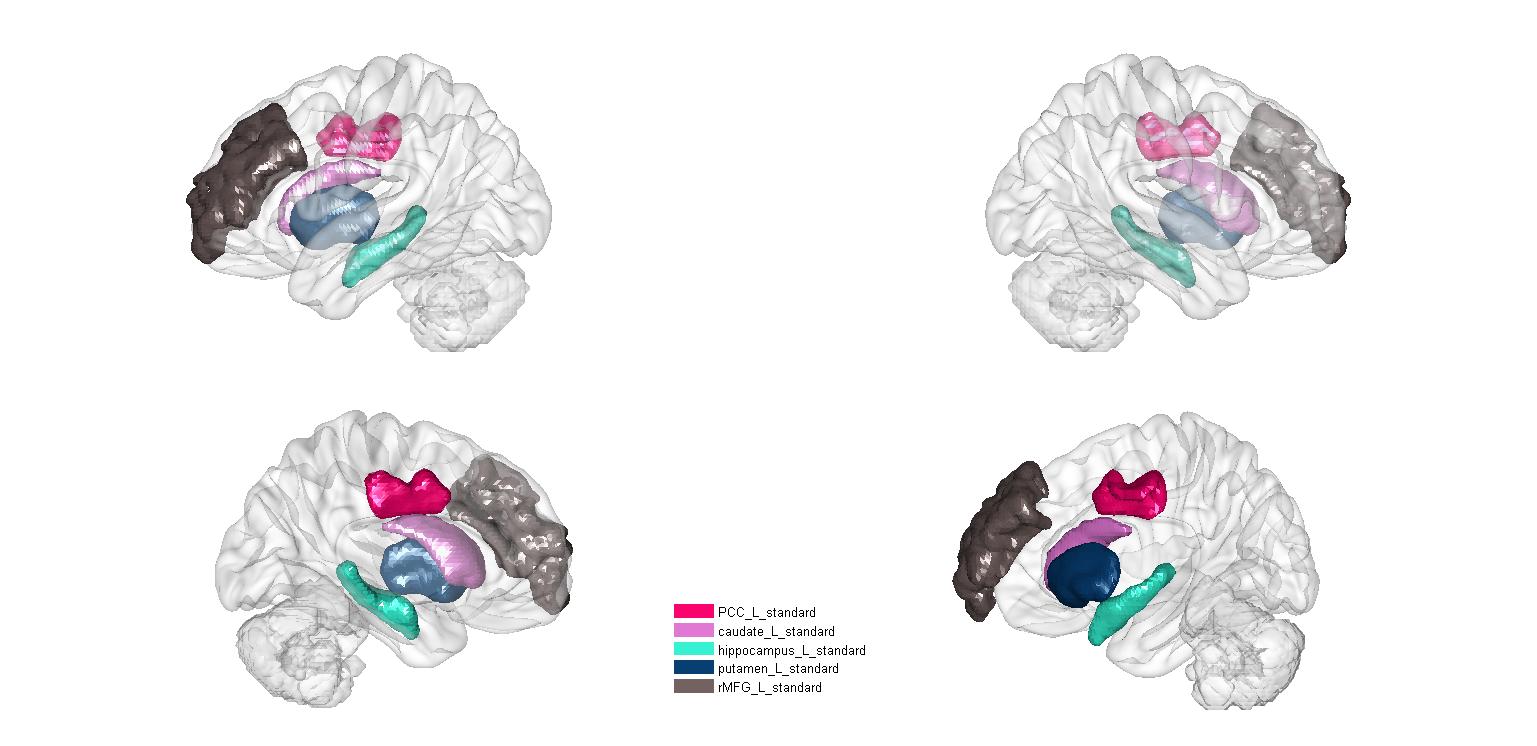

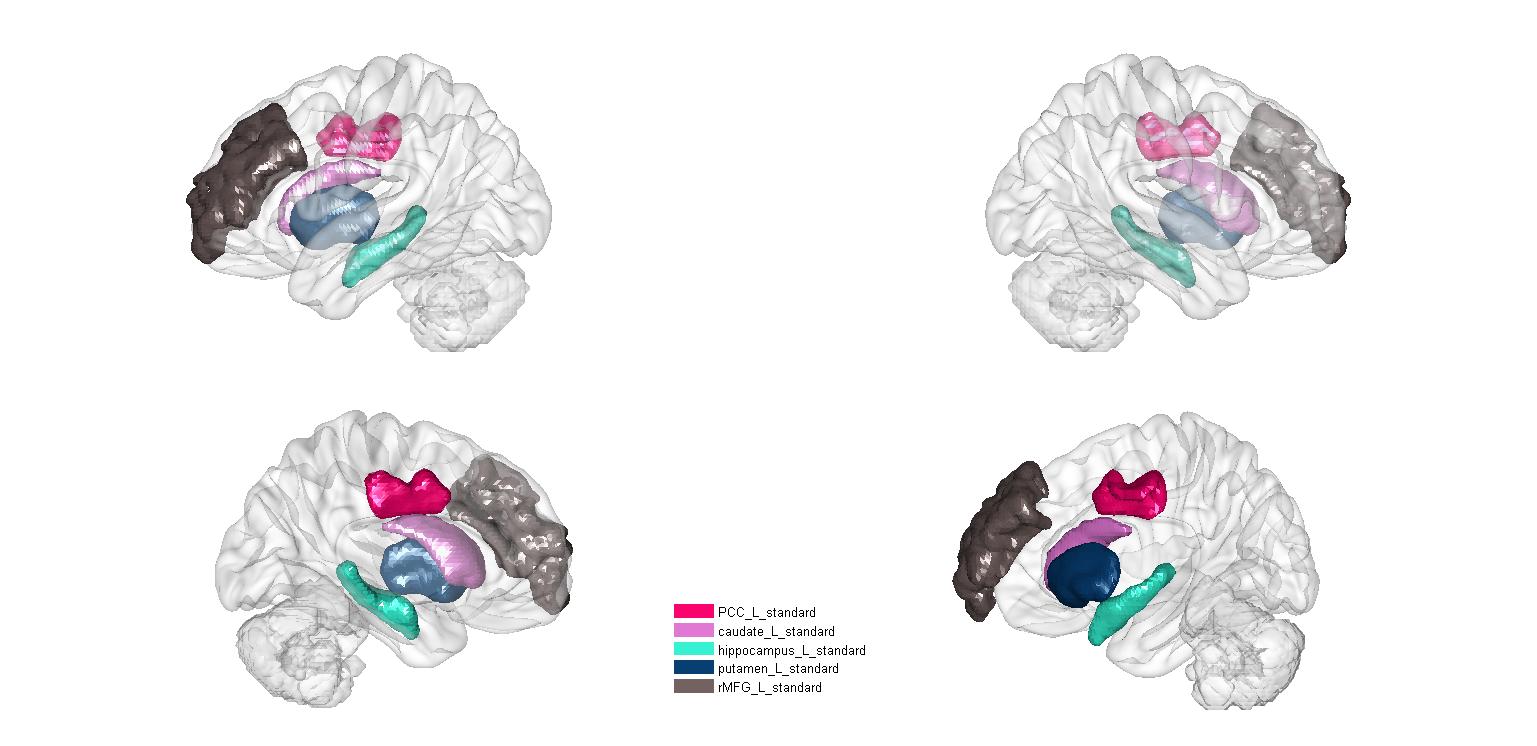

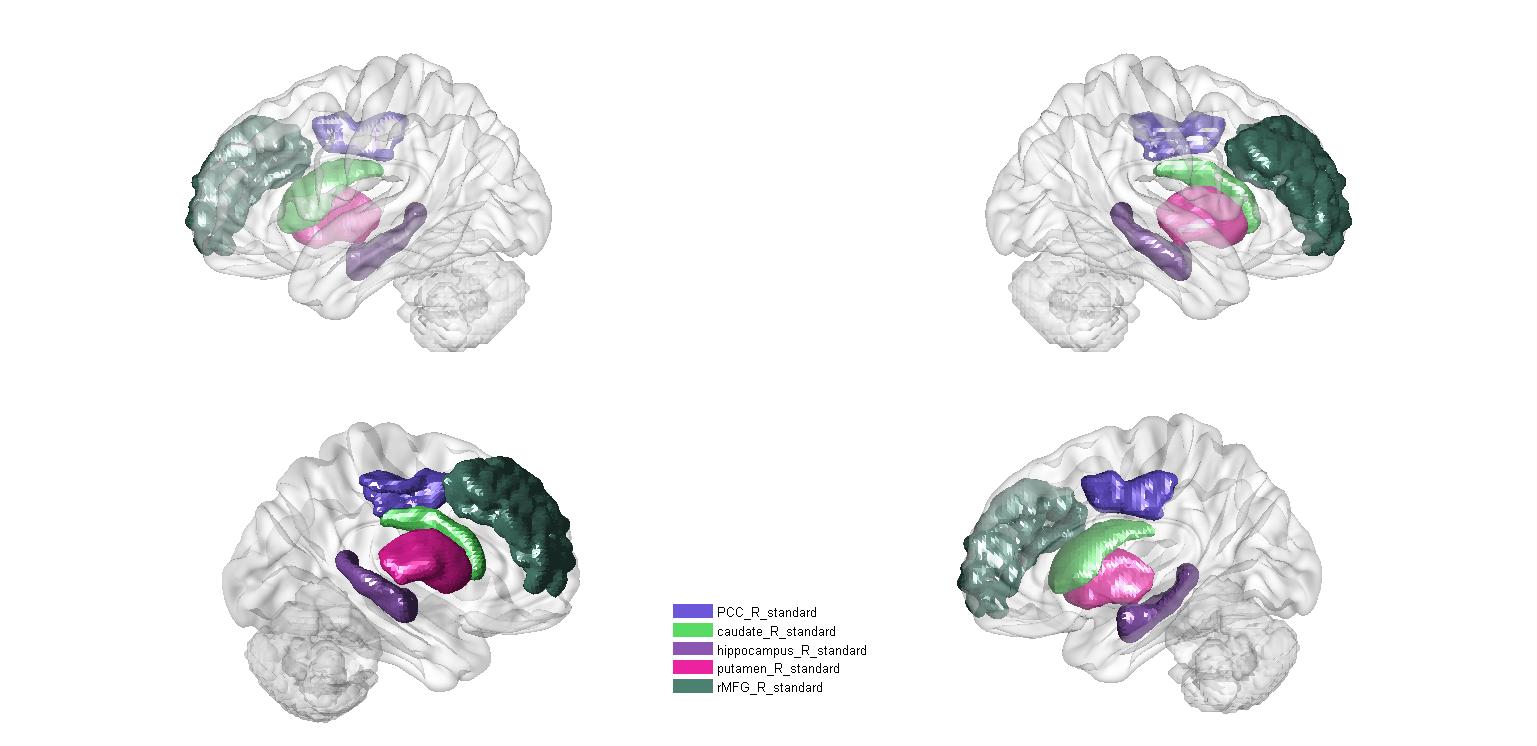

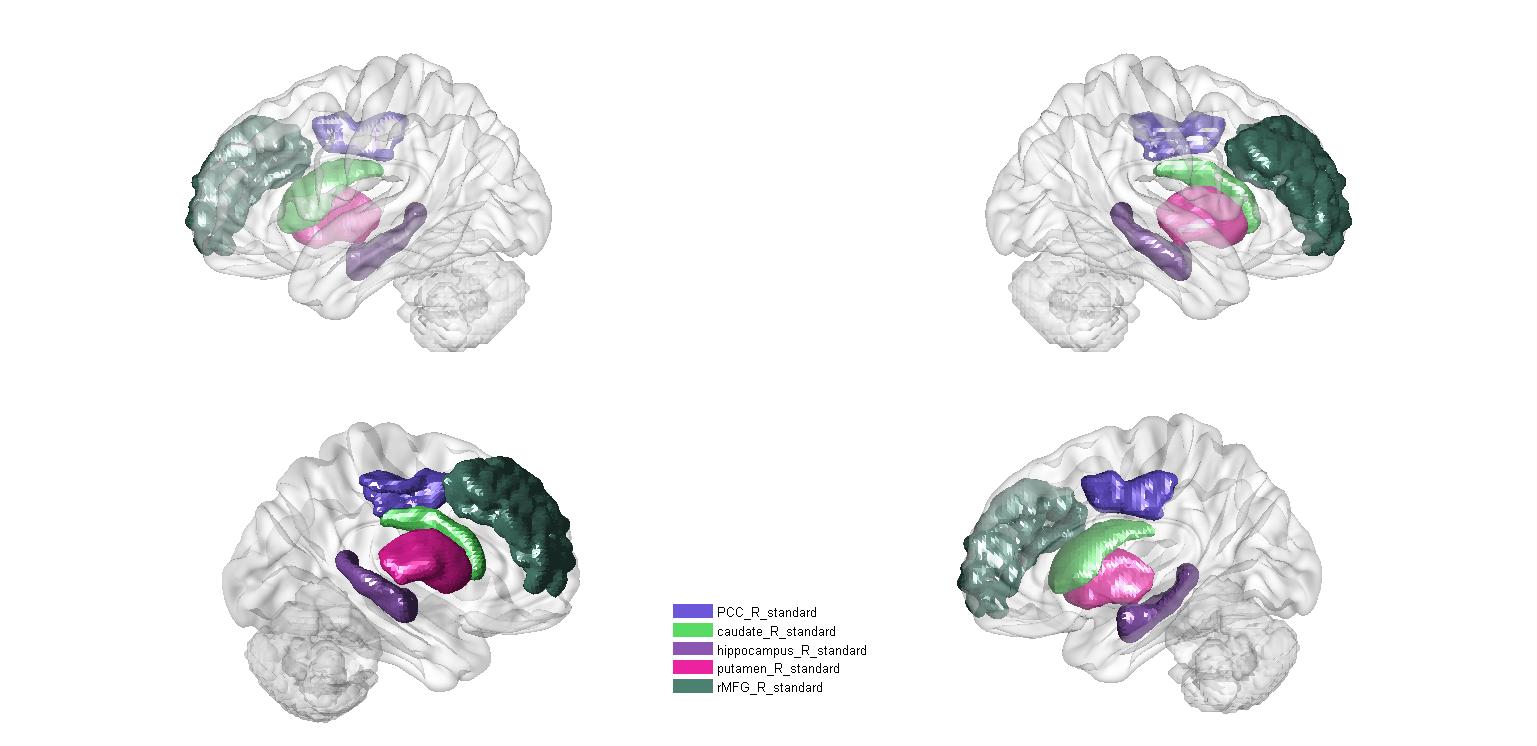


**A**

**B**

**C**

**D**

The standard space template we used is MNI152 template. The ROIs on the left and right hemispheres are displayed from lateral (A-B) and medial (C-D) views, respectively. L = left; R = right.

**Figure S2. Neuropsychological and clinical correlations of the structural and functional connectivity of neural circuits.
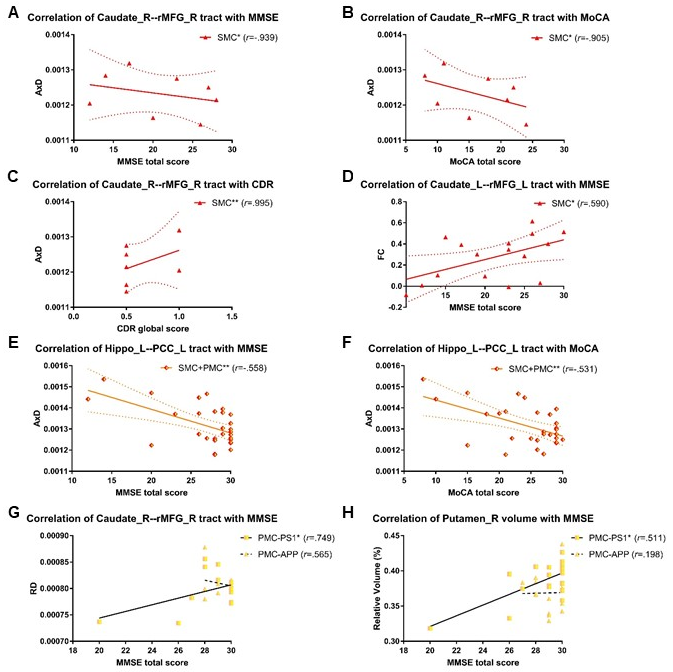
**

Neuropsychological measures include MMSE and MoCA total score; clinical measure is CDR global score. Partial correlations controlling for age, sex and education were performed, in SMC (A-D), all mutation carriers (E-F), and different gene mutation groups of PMC (G-H). The best-linear-fit regression lines with the 95% confidence intervals (dotted curves) are displayed for the convenience of readers. * 0.01 < P < 0.05, ** 0.001 < P < 0.01.

**Figure S3. Correlations of the ROI volume with MMSE.**


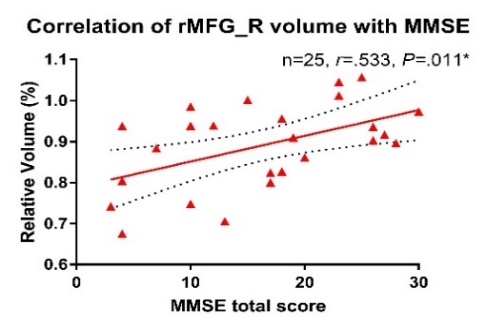

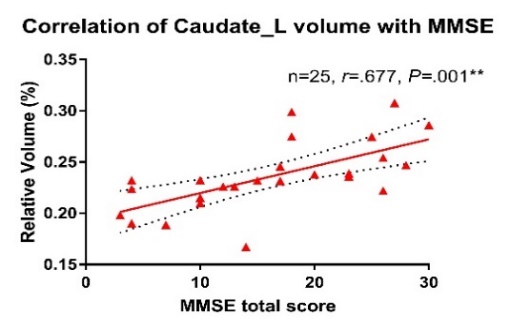

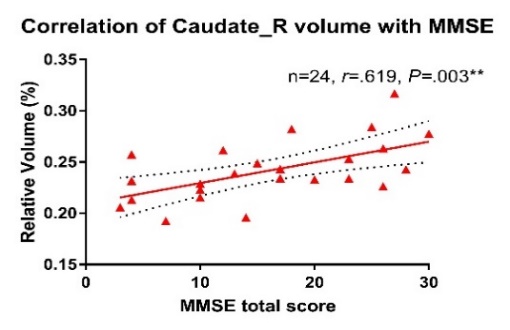

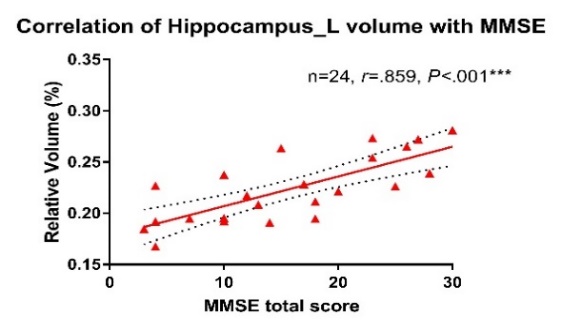

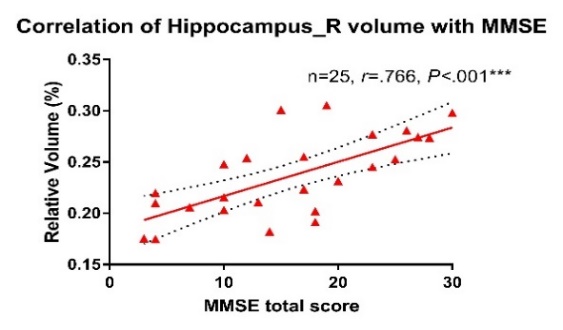

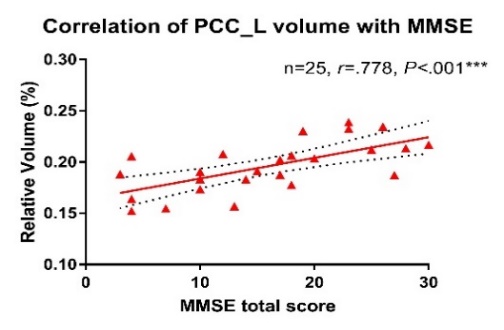

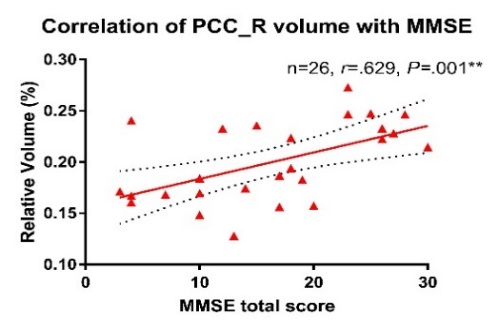

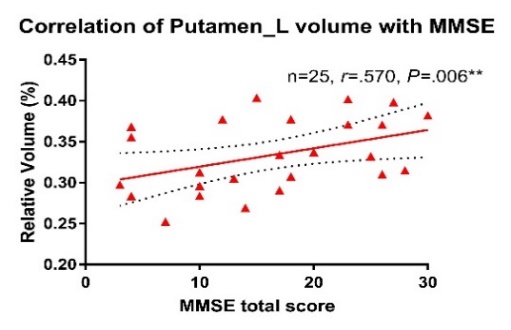

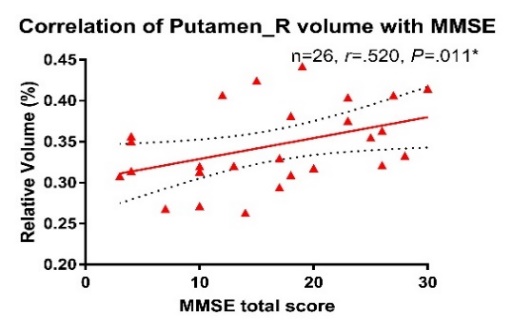

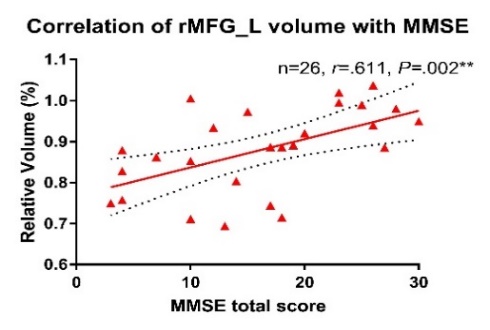


**A**

**B**

**C**

**D**

**E**

**F**

**G**

**H**

**I**

**J**

Partial correlations controlling for age, sex and education were performed in SMC group. The best-linear-fit regression lines with the 95% confidence intervals (dotted curves) are displayed for the convenience of readers. * 0.01 < P < 0.05, ** 0.001 < P < 0.01, ***P < 0.001.

**Figure S4. Correlations of the ROI volume with MoCA.**


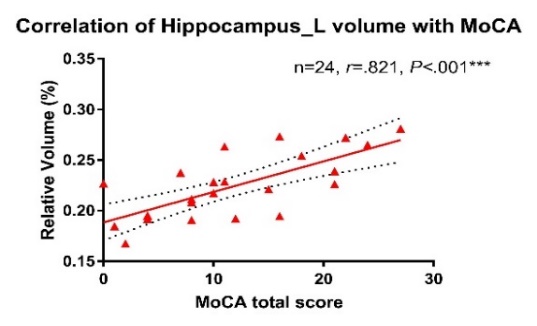

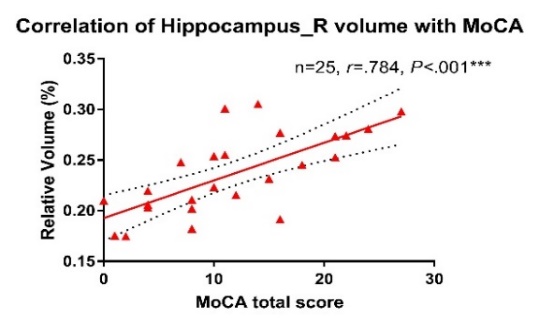

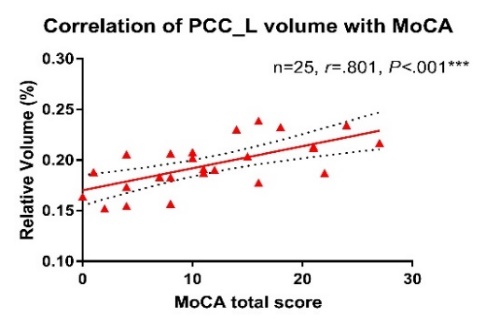

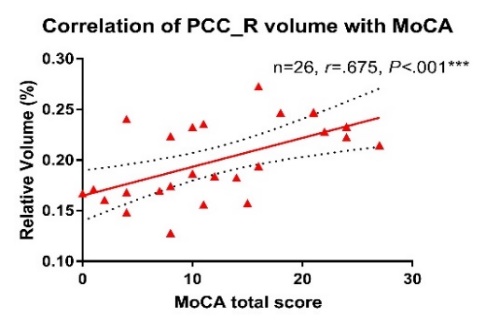

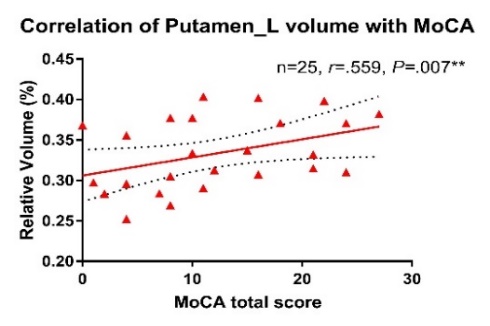

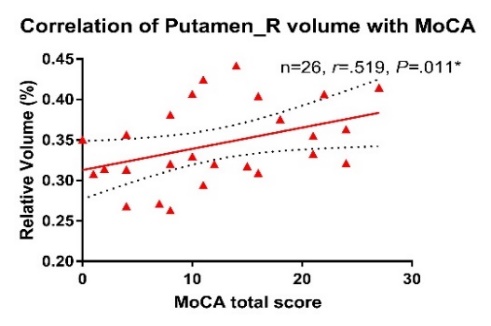

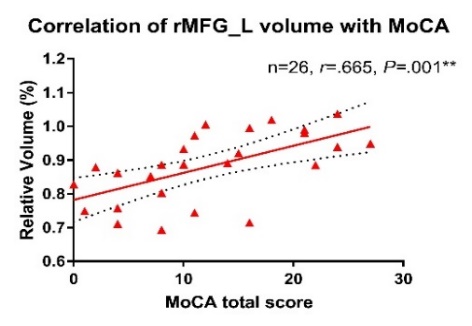

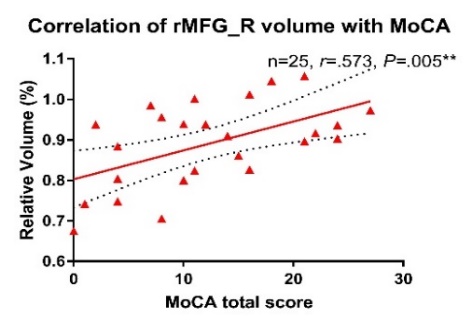

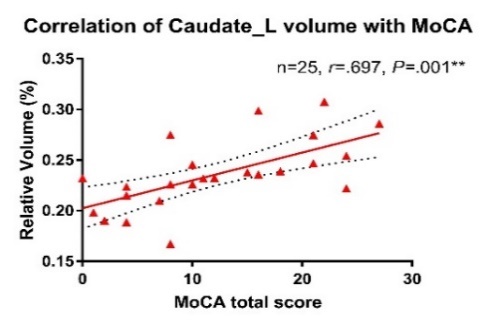

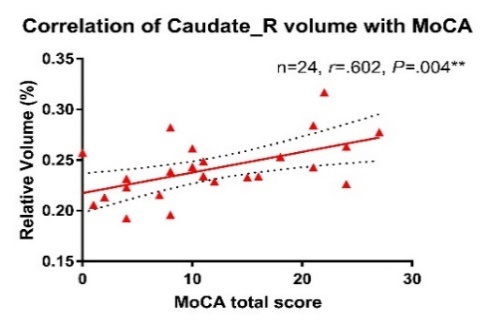


**A**

**C**

**E**

**B**

**D**

**F**

**G**

**H**

**I**

**J**

Partial correlations controlling for age, sex and education were performed in SMC group. The best-linear-fit regression lines with the 95% confidence intervals (dotted curves) are displayed for the convenience of readers. * 0.01 < P < 0.05, ** 0.001 < P < 0.01, ***P < 0.001.

**Figure S5. Correlations of the ROI volume with CDR.**


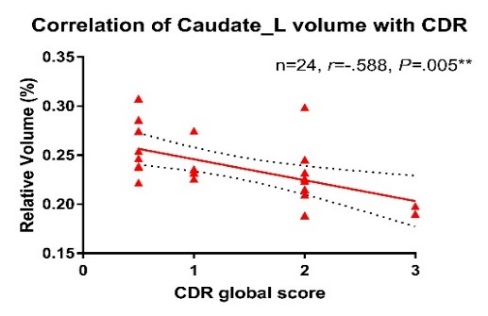

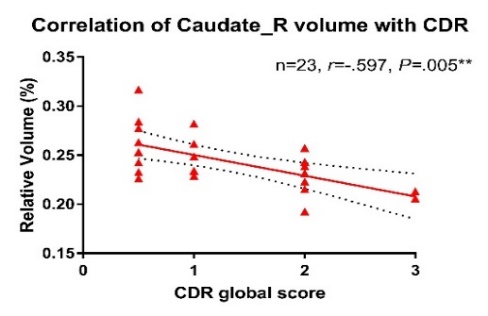

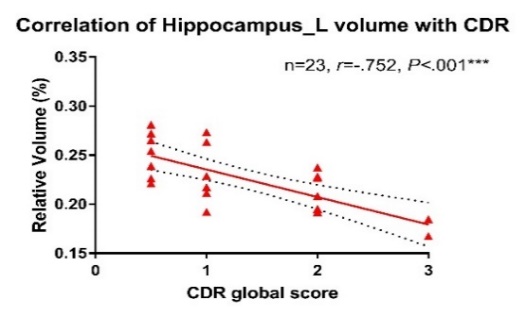

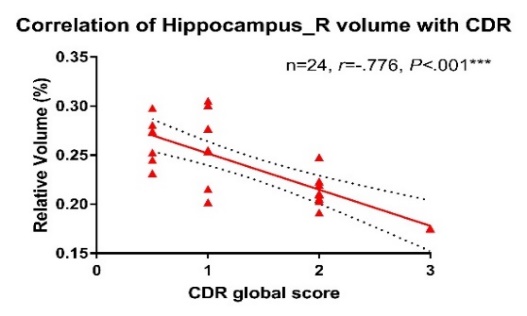

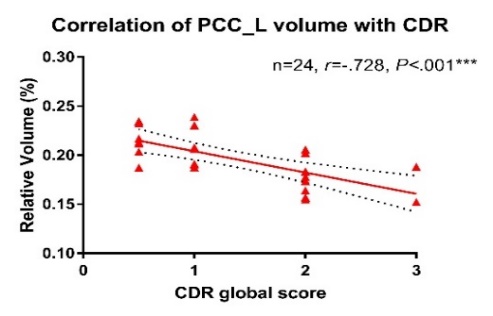

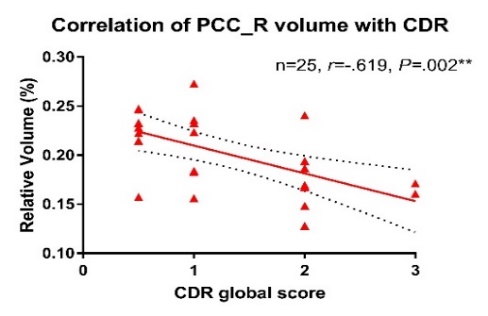

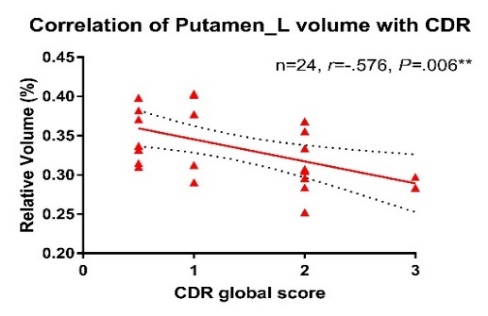

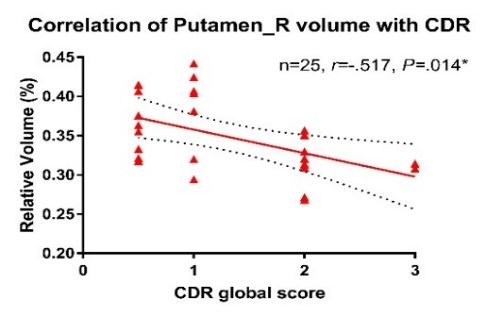

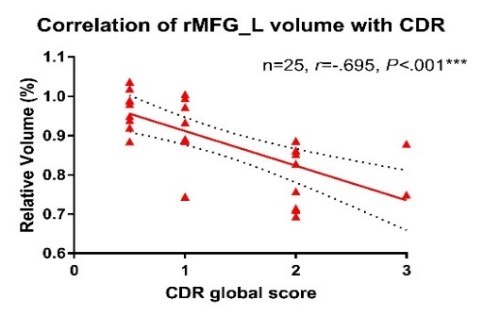

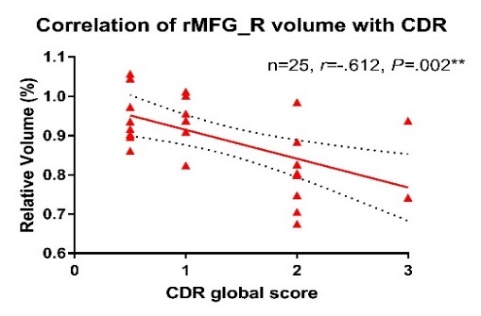


**A**

**C**

**E**

**B**

**D**

**F**

**G**

**H**

**I**

**J**

Partial correlations controlling for age, sex and education were performed in SMC group. The best-linear-fit regression lines with the 95% confidence intervals (dotted curves) are displayed for the convenience of readers. * 0.01 < P < 0.05, ** 0.001 < P < 0.01, ***P < 0.001.

**References:**

1. Fischl B, Salat DH, Busa E, Albert M, Dieterich M, Haselgrove C, van der Kouwe A, Killiany R, Kennedy D, Klaveness S, et al. Whole brain segmentation: automated labeling of neuroanatomical structures in the human brain. Neuron. 2002;333:341-55.

2. Behrens TE, Berg HJ, Jbabdi S, Rushworth MF, Woolrich MW. Probabilistic diffusion tractography with multiple fibre orientations: What can we gain? Neuroimage. 2007;341:144-55.

3. Greve DN, Fischl B. Accurate and robust brain image alignment using boundary-based registration. Neuroimage. 2009;481:63-72.

4. Ashburner J. SPM: a history. Neuroimage. 2012;622:791-800.

5. Xu K, Liu Y, Zhan Y, Ren J, Jiang T. BRANT: A Versatile and Extendable Resting-State fMRI Toolkit. Front Neuroinform. 2018;12:52.
